# An SMC-like protein binds and regulates *Caenorhabditis elegans* condensins

**DOI:** 10.1371/journal.pgen.1006614

**Published:** 2017-03-16

**Authors:** Lucy Fang-I Chao, Meha Singh, James Thompson, John R. Yates, Kirsten A. Hagstrom

**Affiliations:** 1 Program in Molecular Medicine, University of Massachusetts Medical School, Worcester, MA, United States of America; 2 Department of Chemical Physiology, The Scripps Research Institute, La Jolla, CA, United States of America; 3 Department of Biology, College of the Holy Cross, Worcester, MA, United States of America; Emory University, UNITED STATES

## Abstract

Structural Maintenance of Chromosomes (SMC) family proteins participate in multisubunit complexes that govern chromosome structure and dynamics. SMC-containing condensin complexes create chromosome topologies essential for mitosis/meiosis, gene expression, recombination, and repair. Many eukaryotes have two condensin complexes (I and II); *C*. *elegans* has three (I, II, and the X-chromosome specialized condensin I^DC^) and their regulation is poorly understood. Here we identify a novel SMC-like protein, SMCL-1, that binds to *C*. *elegans* condensin SMC subunits, and modulates condensin functions. Consistent with a possible role as a negative regulator, loss of SMCL-1 partially rescued the lethal and sterile phenotypes of a hypomorphic condensin mutant, while over-expression of SMCL-1 caused lethality, chromosome mis-segregation, and disruption of condensin I^DC^ localization on X chromosomes. Unlike canonical SMC proteins, SMCL-1 lacks hinge and coil domains, and its ATPase domain lacks conserved amino acids required for ATP hydrolysis, leading to the speculation that it may inhibit condensin ATPase activity. SMCL-1 homologs are apparent only in the subset of *Caenorhabditis* species in which the condensin I and II subunit SMC-4 duplicated to create the condensin I^DC^- specific subunit DPY-27, suggesting that SMCL-1 helps this lineage cope with the regulatory challenges imposed by evolution of a third condensin complex. Our findings uncover a new regulator of condensins and highlight how the duplication and divergence of SMC complex components in various lineages has created new proteins with diverse functions in chromosome dynamics.

## Author summary

The structure of chromosomes is dynamically modulated to ensure that the genetic material is properly used and passed on to new cells. Evolutionarily conserved proteins of the Structural Maintenance of Chromosomes (SMC) family bind to chromosomes and organize their structure. Through gene duplication and divergence, eukaryotes have evolved multiple SMC-containing protein complexes that handle different aspects of genome organization. In this study, we identify a novel SMC-like protein that binds to conserved SMC proteins within *Caenorhabditis elegans* condensin complexes. This SMC-like protein has an atypical truncated structure and may act as a regulator to restrict the chromosome separation and gene regulation activities of condensin complexes. Similar SMC-like proteins are encoded only in a related subset of *Caenorhabditis* species, and these species also have a duplicated condensin SMC protein, suggesting co-evolution of a condensin regulator along with an additional condensin complex. Thus, we describe a new regulator of SMC complexes that is itself SMC-like, highlighting how the evolutionary divergence of this important protein family has allowed organisms to effectively utilize and safeguard their genomes.

## Introduction

Chromosome organization influences genome function. Condensins are conserved protein complexes that reconfigure chromosome architecture to promote chromosome segregation, recombination, DNA repair, and gene expression regulation (reviewed in [1–5]). Condensins drive ATP-dependent supercoiling, compaction, and entrapment of DNA strands [6]. Across the three domains of life, condensins compact and segregate chromosomes during cell division. Condensins also organize chromosome topology during interphase and are implicated in a variety of organism- and cell type-specific aspects of chromosome function such as gene regulation. Consistent with diverse important roles in chromosome dynamics, loss of condensin function *in vivo* causes severe phenotypes and often lethality.

Eukaryotic condensins are five-subunit complexes containing a heterodimer pair of SMC (Structural Maintenance of Chromosomes) subunits and three CAP (Chromosome-Associated Proteins) subunits. SMC proteins are a family of ATPases with critical functions in chromosome dynamics from bacteria to humans. SMC proteins fold back on themselves and dimerize to form functional ATPases. The cycle of ATP binding, ATP-dependent contact between ATPase domains of two SMC proteins, and hydrolysis is essential for the chromosome binding and functions of SMC complexes, including condensin [2, 5]. The condensin subunit CAP-H is a kleisin (closure) protein that contacts each SMC subunit, forming a closed ring that is proposed to encircle and topologically link distant DNA regions during chromosome condensation [7–10]. The other CAP subunits are the HEAT-repeat containing proteins CAP-D and CAP-G, which interact with the kleisin subunit to complete the functional five-subunit complex [8]. DNA binding activity has been described for condensin SMC proteins [11, 12] and for its HEAT-containing CAP subunits [13].

Most eukaryotes have two related condensin complexes, condensins I and II. They each contain the same SMC2/SMC4 dimer pair, but differ in their sets of paralogous CAP subunits. Condensin I uses CAP-D2, CAP-G, and CAP-H and condensin II uses CAP-D3, CAP-G2, and CAP-H2 [14, 15]. Although the two complexes partially overlap in subunit composition, they have non-overlapping functions [2]. Condensins I and II differ in some of their binding sites on chromosomes, cell cycle timing of binding, and ancillary factors required for binding [4, 14–21]. During mitosis, condensin I compacts chromosomes along their width, while condensin II performs longitudinal compaction [14, 22–24]. Depletion of subunits unique to condensin I or II causes different phenotypes, and depletion of both is often more severe [14, 16, 17, 24].

A key question in chromosome biology is how condensin complexes are regulated to create spatially and temporally appropriate chromosome structures. Studies have revealed layers of both positive and negative regulation of condensins, which can be divided into three major modes: regulation of condensin activity, chromosome binding, or abundance. Condensin activity is controlled by a number of post-translational modifications [4]. For example, a network of kinases and phosphatases act at different cell cycle phases to ensure that condensins have distinct activities during mitosis and interphase. Each condensin complex is controlled by factors that promote or prevent binding to target sites on chromosomes. Factors promoting binding include specific DNA sequence motifs, protein cofactors, and histone variants and modifications (e.g. [18, 19, 25–30]. Factors inhibiting binding include the human MCPH1 protein, which prevents condensin II binding [31, 32], and histone H3 acetylated on lysine 56, which repels the single fission yeast condensin from chromatin [33]. Condensins are also regulated at the level of abundance. Properly regulated degradation of condensin II and an appropriate ratio of condensin I to condensin II are critical for proper chromosome shape [23, 24, 34, 35]. The multiple condensin regulatory mechanisms must cooperate to ensure that condensins build chromosome structures appropriate to particular chromosome regions and functions, to the phase of the cell cycle, and to the developmental stage and cell type.

**Fig 1:**
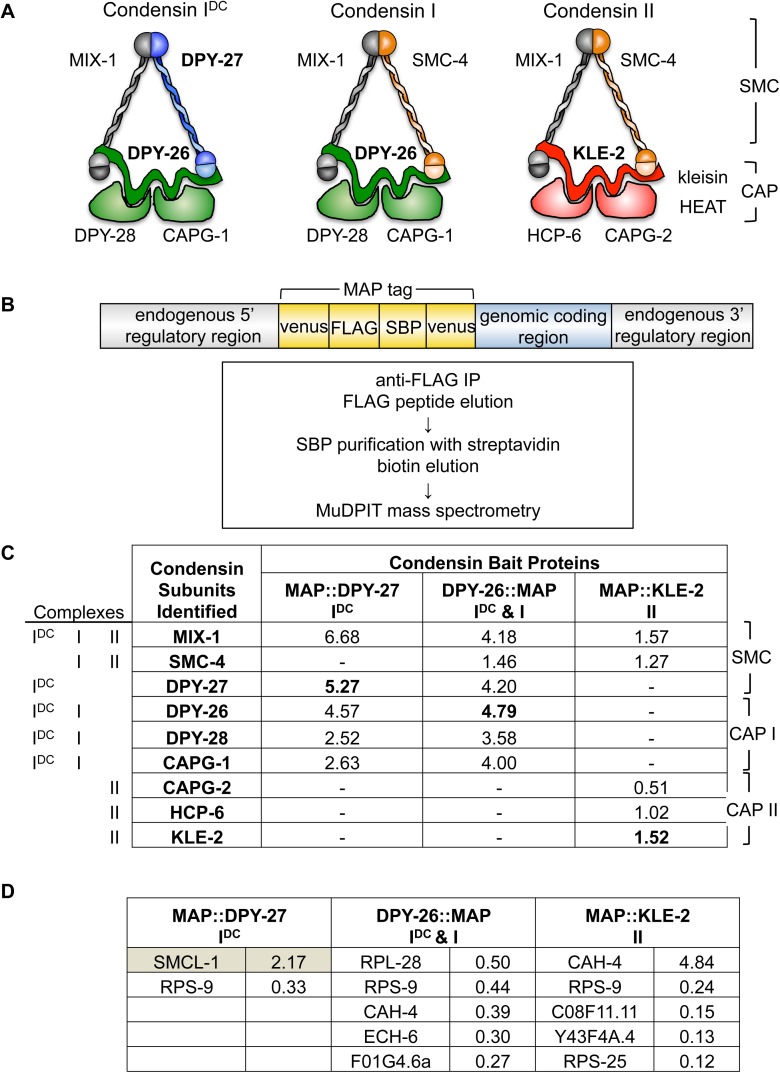
Identification of SMCL-1 as a novel protein that co-purifies with condensin subunits. (A) The three condensin complexes in *C*. *elegans*. Subunits used for purification in bold. (B) MAP-tagged condensin transgenes and strategy for tandem affinity purification and protein identification. DPY-27 and KLE-2 were tagged at the N-terminus; DPY-26 was tagged at the C-terminus. The MAP tag (yellow) includes mVenus for visualization, and the FLAG epitope and SBP for tandem affinity purification. (C-D) Identities of (C) condensin proteins and (D) non-condensin proteins that co-purified during tandem affinity purification from adult extracts with MAP-tagged DPY-27, DPY-26, or KLE-2 but not in untagged control, and were identified by MudPIT mass spectrometry. Relative protein abundance in the sample was estimated by calculating the number of spectra identified, normalizing it to protein length, and dividing by the total spectra in the sample. These normalized spectral abundance factor (NSAF) values are shown as the average from two replicas. The non-condensin proteins with the five highest NSAF values are shown in D; SMCL-1 is highlighted. All other mass spectrometry data in S1–S10 Tables.

The nematode *C*. *elegans* has a third condensin complex, making this organism useful for investigating the evolution and regulation of multiple condensin complexes. In addition to condensins I and II, *C*. *elegans* has condensin I^DC^, a complex that shares all but one subunit with condensin I but has a different function (Fig 1A, [16]). Condensins I and II condense all mitotic/meiotic chromosomes to promote their segregation. Condensin I^DC^, along with additional proteins that form a larger dosage compensation complex, restructures the two X chromosomes of hermaphrodites to down-regulate X-linked gene transcription and equalize transcript doses with those from the single X in XO males ([36] and reviewed in [37–39]). Whereas condensin I and II bind along all chromosomes, condensin I^DC^ concentrates onto the two X chromosomes. A few factors have been identified that influence binding of each *C*. *elegans* condensin. Binding of condensin I^DC^ to hermaphrodite X chromosomes is promoted by X-enriched DNA sequence motifs [18, 28, 40, 41], by the other subunits of the complete dosage compensation complex and sumoylation of several of these proteins [42], and is restricted by the histone variant H2A.z [43]. All three condensins require the SDC-2 and SCC-2 proteins for their binding on X chromosomes [18]. Targeting of condensin I, but not II, to chromosomes requires the Aurora B kinase [44, 45]. Despite these discoveries, the mechanisms that regulate three *C*. *elegans* condensin complexes with partially overlapping subunits remain largely unknown.

We reasoned that many regulatory mechanisms would involve association between a regulatory protein and condensin, and set out to identify proteins that co-purify with *C*. *elegans* condensin subunits. Here we report the identification of SMCL-1, a previously uncharacterized protein that associates with *C*. *elegans* condensin subunits. SMCL-1 co-purified with condensin SMC subunits, and itself resembles SMC family proteins, but lacks domains critical for SMC protein function. Loss of SMCL-1 showed no obvious phenotype. However, consistent with a potential role as a negative regulator, loss of SMCL-1 restored greater viability and fertility to a hypomorphic condensin mutant, while overexpression of SMCL-1 caused sterility, lethality, and chromosome segregation defects. Moreover, overexpression of SMCL-1 disrupted condensin I^DC^ localization to X chromosomes. Homologs of SMCL-1 exist only in a subset of *Caenorhabditis* species that overlaps with the lineage in which homologs of both SMC-4 and the related condensin I^DC^-specific DPY-27 protein are detected. We propose that SMCL-1 became adapted as a regulator to accommodate regulatory disturbances imposed by the evolution of the specialized condensin I^DC^ complex in a recent *Caenorhabditis* lineage. Thus the duplication and divergence of SMC complex subunits has led not only to the creation of multiple complexes with varied functions, but also to the adaptation of an SMC-like protein for a regulatory role.

## Results

### Identification of condensin-interacting proteins

*C*. *elegans* harbors three condensin complexes; each has five subunits and they partially overlap in subunit composition, but differ in chromosomal localization and function (Fig 1A)[16]. To better understand how the three condensin complexes achieve their localization and functions, we identified proteins associated with a tagged “bait” subunit representative of each complex. DPY-27 (I^DC^), DPY-26 (I & I^DC^) and KLE-2 (II) were each fused to the Multifunctional tandem Affinity Purification (MAP) tag that includes the fluorescent protein mVenus, the FLAG epitope, and the Streptavidin Binding Peptide (SBP) [46] (Fig 1B). Resulting single-copy transgenic strains expressed fusion proteins that rescued respective null mutants and localized properly on chromosomes (S1 Fig). Tandem affinity purification from adult hermaphrodite extracts was performed (Fig 1B), co- purified proteins were identified by MudPIT mass spectrometry, and proteins identified in two replicates and not in an untagged wild-type control sample were ranked by their Normalized Spectral Abundance Factor (NSAF) values [47] (see Methods, S1–S4 Tables). Consistent with our purifications of untagged condensin subunits reported previously [14], purification of MAP::DPY-27 recovered condensin I^DC^ subunits and purification of MAP::KLE-2 recovered condensin II subunits, while purification of DPY-26::MAP recovered subunits of both the condensin I and I^DC^ complexes in which it participates (Fig 1C). For all three subunits purified, known condensin complex members were the highest scoring interacting proteins, confirming the effectiveness of our method (S1–S3 Tables).

We examined the highest-ranking non-condensin proteins that co-purified with each tagged subunit (Fig 1D). Several are likely contaminants that remain despite our strategy, such as the carbonic anhydrase enzyme CAH-4 and ribosomal subunits (e.g. RPS-9), which are common contaminants in affinity purifications [48]. But one protein, encoded by the gene C44C10.4, stood out as an interesting condensin-interacting protein for several reasons: it scored the highest among non-condensin co-purified proteins, it co-purified strongly with MAP::DPY-27 but not with DPY-26::MAP or MAP::KLE-2, and it resembles the SMC family of proteins (more details below), which includes the core condensin SMC subunits. We therefore named the C44C10.4 protein SMCL-1, for Structural Maintenance of Chromosomes Like-1, and decided to pursue this novel condensin-interacting protein.

### SMCL-1 interacts with multiple condensin subunits

**Fig 2:**
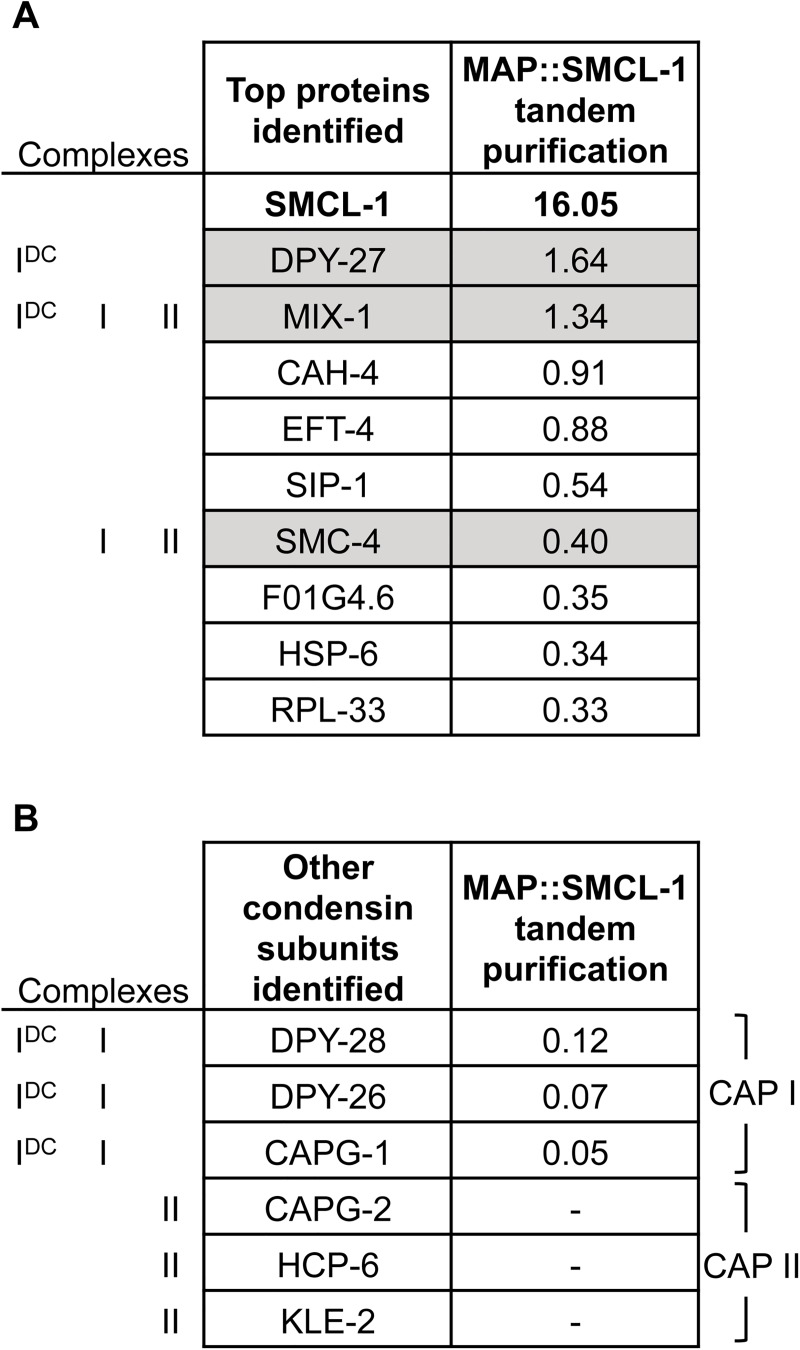
Condensin subunits co-purify with MAP::SMCL-1. (A-B) Proteins that co-purified with MAP::SMCL-1 but not untagged control adult extracts, identified by tandem affinity purification and MudPIT mass spectrometry. Numbers represent average NSAF values from two replicas. Co-purified proteins with the highest NSAF values are shown in (A), values for other condensin subunits are shown in (B), and all other proteins are shown in S5 Table. Condensin SMC subunits are highlighted.

We wondered whether the SMCL-1 protein interacted exclusively with DPY-27, or associated with other condensin subunits, other SMC proteins, or other complexes. We generated a MAP-tagged SMCL-1 transgenic strain and performed tandem purification followed by MudPIT mass spectrometry (Fig 2A and 2B, S5 Table).

We made several observations from our MAP::SMCL-1 purification. First, among all proteins that co-purified with MAP::SMCL-1, the three condensin SMC subunits DPY-27, MIX-1, and SMC-4 were among the top interactors with some of the highest NSAF values (1.64, 1.34, and 0.40, respectively, Fig 2A, S5 Table). Since DPY-27 and SMC-4 do not exist in a common complex (Fig 1C and [16, 49, 50]), we speculate that SMCL-1 may interact with individual SMC proteins to form dimers or with SMC dimer pairs to form trimers. Second, CAP subunits of condensin II were not recovered at all and CAP subunits shared between condensins I^DC^ and I were recovered with lower NSAF values (0.09–0.21) compared to SMC subunits (Fig 2B). This suggests that SMCL-1 is not an obligate member of a complete five-subunit functional condensin complex. We did recover low levels of DPY-26 in our MAP::SMCL-1 purification whereas SMCL-1 was not present in our initial MAP::DPY-26 purification. A potential explanation is that the two proteins interact at a low level and since SMCL-1 is less abundant than DPY-26 (S2 Fig), purification with MAP::SMCL-1 enriches for this interaction to allow easier identification through MudPIT. Third, purification of MAP::SMCL-1 yielded more peptides (higher NSAF value) corresponding to SMCL-1 than to condensin subunits (Fig 2A and 2B). While our experiments cannot quantitatively assess stoichiometry, this finding suggests that there may be a pool of SMCL-1 free from condensin subunits, or that its interaction with condensin subunits was unstable during purification. Finally, SMCL-1 did not co-purify subunits of other SMC-containing complexes (cohesin and the repair SMC5/6 complex), with the exception of the cohesin subunit HIM-1 at a very low NSAF value (0.02, S5 Table). This finding suggests that SMCL-1 function is related to condensins but not to other SMC complexes.

Together, these data suggest that SMCL-1 interacts with condensin proteins but behaves differently from a canonical condensin subunit. SMCL-1 interacted with all three condensin SMC subunits, even the two SMC4 variants that participate in exclusive complexes, SMCL-1 showed greater interaction with SMC than with CAP subunits, and SMCL-1 weakly co-purified CAP I but not any CAP II subunits. We therefore hypothesize that SMCL-1 is not a full-time member of a single five-subunit condensin holocomplex, but instead may bind to condensin SMC proteins to serve another role, such as condensin regulation.

### Characterization of *smcl-1* gene expression

**Fig 3:**
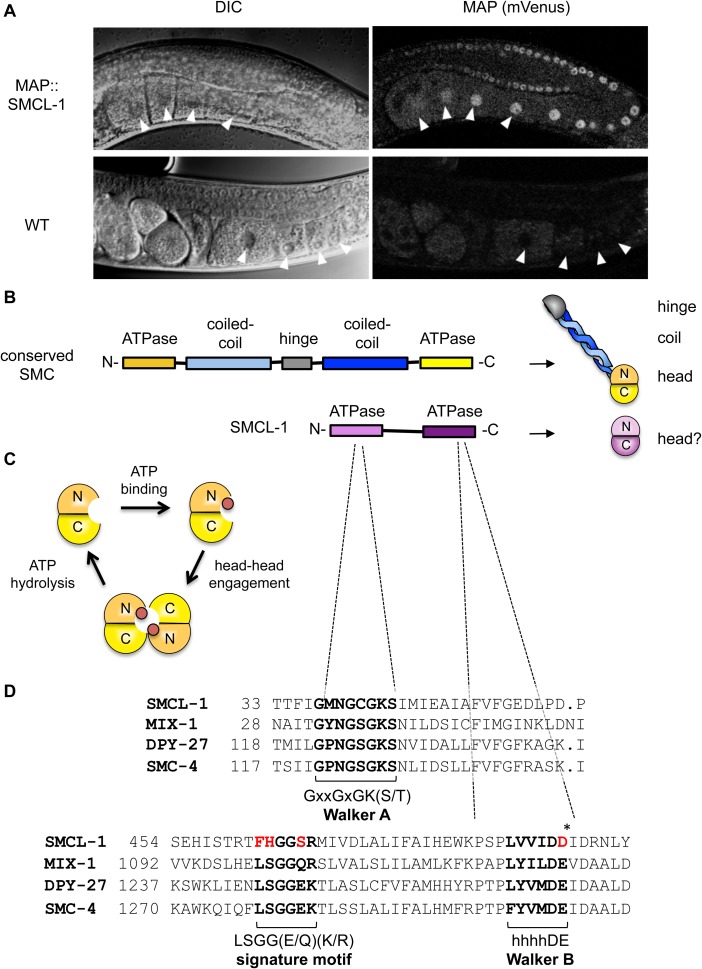
SMCL-1 expression and protein features. (A) Adult hermaphrodites from wild-type (WT) and a strain carrying the *map*::*smcl-1* transgene driven by endogenous *smcl-1* 5’ and 3’ elements. A section of the germline is shown, imaged by DIC to show structures and fluorescent microscopy to detect mVenus expression from the MAP tag. Arrowheads denote the first four oocytes. (B) A typical SMC protein folds back on itself at a hinge domain, bringing coil regions together and creating a “head domain” (yellow) from ATPase domains in the N- and C-termini. SMCL-1 lacks predicted coil and hinge domains, but has N- and C-terminal ATPase domains that may be capable of forming a head domain (purple). (C) SMC head domain and the ATPase cycle, showing binding of ATP (red circle), ATP-dependent engagement of heads from two SMC proteins, and disengagement upon ATP hydrolysis. (D) SMCL-1 amino acid sequence aligned to *C*. *elegans* condensin SMC proteins. Shown are regions surrounding three conserved motifs found in SMCs and related ATPases: the Walker A motif, ABC transporter signature motif, and Walker B motifs, and their consensus sequences. SMCL-1 shares a conserved Walker A motif, but differs from consensus signature motif and Walker B motif at residues shown in red. Asterisk denotes catalytic amino acid required for ATP hydrolysis. x = any amino acid and h = hydrophobic amino acid.

To evaluate expression of the *smcl-1* gene (C44C10.4), we examined compiled RNA-sequencing data available at Wormbase. These data indicate that *smcl-1* mRNA is expressed throughout development, like condensin subunits, but at lower levels than condensin subunits (S2 Fig). To evaluate SMCL-1 protein expression and subcellular localization, we took advantage of our MAP-tagged *smcl-1* transgene, which is under control of endogenous 5’ and 3’ regulatory elements (Fig 1B). A fluorescent signal from mVenus within the MAP::SMCL-1 fusion protein was only obvious in the germline, where it localized to nuclei (Fig 3A). MAP-tagged SMCL-1 localized in a pan-nuclear pattern, in contrast to the sub-nuclear pattern of condensin I^DC^ to X chromosomes or of condensins I and II to mitotic/meiotic chromosomes [16, 45], which we did observe with MAP-tagged DPY-27, DPY-26, and KLE-2 (S1B Fig). We also raised an antibody against the C-terminus of SMCL-1, and in immunofluorescence experiments the antibody recognized overexpressed SMCL-1 but not the low endogenous levels of SMCL-1 (see below). Thus, the *smcl-1* gene produces low transcript levels throughout development, and a protein most obviously visualized in germline nuclei that does not seem to share the distinctive X-chromosome or mitotic/meiotic chromosome-binding patterns of functional condensin subunits.

### SMCL-1 is an SMC-like protein predicted to lack ATPase activity

The SMCL-1 protein is homologous to the SMC family of ATPases, but exhibits key differences that suggest it may not act like a typical SMC protein. SMC proteins share several features in primary structure: a conserved central hinge region flanked by coiled-coil regions, and conserved ATPase motifs at the N- and C- termini (Fig 3B). When each SMC protein folds back on itself at the hinge, anti-parallel coils are juxtaposed and nucleotide binding and hydrolysis domains present in each terminus are brought together to form the ATPase head domain (Fig 3B). When SMC proteins heterodimerize, they interact through their hinge and coiled-coil domains, and these domains provide the specificity and strength for dimer formation, respectively. When the ATPase head domain of each SMC of the heterodimer binds ATP, the heads come together (“engage”) with 2 ATP molecules sandwiched between them (Fig 3C), creating a closed ring structure, in addition to the ring created by the attachment of the kleisin subunit to the two different SMC heads. The ATPase head domains disengage upon ATP hydrolysis (Fig 1A depicts disengaged heads and the kleisin connection). The ATPase cycle is required for proper condensin loading, dynamics, and function, likely by allowing the condensin complex ring to topologically entrap DNA [10, 51–54].

SMCL-1 differs from canonical SMC proteins in two major ways. First, SMCL-1 retains the N- and C-terminal ATPase domains characteristic of SMC proteins, but lacks the conserved hinge and predicted coiled-coil domains (Fig 3B, S3 Fig). The observed interaction between SMCL-1 and the SMC2 and SMC4 class proteins thus cannot occur via hinge-to-hinge interactions, but instead likely involves the ATPase domains of SMCL-1, which may fold into an ATPase head domain. Second, while SMCL-1 has the conserved Walker A motif required for ATP binding, SMCL-1 lacks conserved amino acids in the signature motif required for canonical head engagement, and lacks the critical catalytic glutamate in the Walker B motif required for ATP hydrolysis (Fig 3D). When mutations similar to the changes observed in these regions of the wild-type SMCL-1 have been engineered into condensin SMC subunits, the holocomplex can still form and bind ATP, but head engagement, ATP hydrolysis, and loading onto chromosomes is disrupted (e.g. [8, 10, 11, 54, 55]). Importantly, when a related ABC transporter ATPase was mutagenized to make the same catalytic glutamate to aspartate change as found in the wild-type SMCL-1, the ATP hydrolysis activity of the transporter was completely destroyed [56]. These findings predict that SMCL-1 may have ATP binding capability but lack ATP hydrolysis ability. The ATP that binds to the head domain of one SMC of a dimer requires an intact ATP hydrolysis domain in the head of the second SMC for its hydrolysis, and accordingly mutant SMC proteins with impaired ATP hydrolysis activity can dominantly inhibit the ATPase activity of wild-type SMCs [52, 57]. This raises the possibility that SMCL-1 inhibits the ATPase activity of the SMC proteins it contacts.

Based on the unusual structure of SMCL-1 and prior studies of ATPase mechanisms, we hypothesize that when SMCL-1 binds to condensin SMC proteins, it interferes with steps of the ATPase cycle, thereby impairing condensin function. Our finding that SMCL-1 showed greater co-purification of SMC subunits than CAP subunits implies that SMCL-1 could additionally impair condensin function by sequestering SMC subunits and preventing the formation of functional five-subunit holocomplexes.

### SMCL-1 deletion partially suppresses the lethality and sterility defects of a *dpy-28* mutant

To test the hypothesis that SMCL-1 regulates condensin complexes, we created strains that 1) produce no SMCL-1 protein or 2) produce excess SMCL-1 protein. While our data thus far did not support a positive regulator model, it remained possible that SMCL-1 promotes condensin function, in which case the loss of SMCL-1 would be predicted to cause condensin loss-of-function phenotypes and to enhance defects of condensin hypomorphic mutants. In contrast, if SMCL-1 inhibits condensin function, then we predicted that the loss SMCL-1 would suppress defects of condensin hypomorphic mutants while excess SMCL-1 would cause condensin loss-of-function phenotypes.

**Fig 4:**
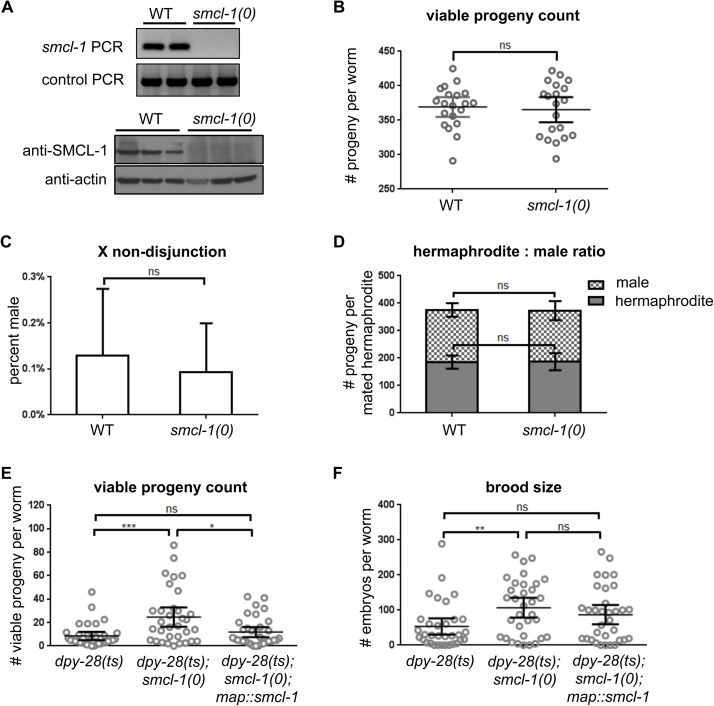
*smcl-1* deletion does not cause condensin loss-of-function phenotypes, but can partially suppress lethality and sterility of a *dpy-28* mutant. (A) PCR and Western blot analyses confirming genomic deletion of *smcl-1* by a MosDEL strategy (see Methods). (B-D) *smcl-1(0)* worms were assayed for phenotypes associated with condensin loss-of-function. (B) Viable progeny count: The number of total viable progeny produced throughout the lifetime of a hermaphrodite was counted from 20 wild-type and 20 *smcl-1(0)* worms. (C) X nondisjunction: Hermaphrodite and male progeny of unmated wild-type or *smcl-1(0)* hermaphrodite worms were counted to assay for an increase in spontaneous production of males, which indicates mis-segregation of the X chromosome. ~6,600 progeny were counted from each strain in four trials. (D) Hermaphrodite vs. male ratio: Hermaphrodite and male progeny of 10 mated wild-type or *smcl-1(0)* hermaphrodite worms were counted to assay for hermaphrodite-specific lethality. (E-F) The effect of deleting *smcl-1* in the *dpy-28(y1)* hypomorphic, temperature-sensitive (ts) strain was assayed. (E) Viable progeny count: The number of total viable progeny produced throughout the lifetime of the worm was counted from 33 hermaphrodite worms of each specified genotype raised at the semi-permissive temperature of 18°C. (F) Brood size: The number of embryos laid throughout the lifetime of the worm was counted from 33 hermaphrodite worms of each genotype raised at the semi-permissive temperature of 18°C. Non-parametric Mann-Whitney test was used for all statistical analyses. *** denotes p ≤ 0.001; ** denotes p ≤ 0.01; * denotes p ≤0.05; ns = not significant. Bars represent 95% confidence intervals.

We created a null allele (*smcl-1(stn1)*, referred to as *smcl-1(0)* here), by deleting the *smcl-1* open reading frame using the MosDEL targeted gene deletion technique [58] (see Methods). Successful deletion was validated by PCR amplification of genomic DNA and by Western analysis (Fig 4A). Initial observations revealed no obvious phenotypes, so we examined homozygous *smcl-1(0)* worms for specific phenotypes associated with the loss of condensin functions in mitosis, meiosis, and dosage compensation [59]. Brood size and survival of progeny is lower in all condensin subunit mutants compared to wild-type animals. However, the total number progeny that grow to the adult stage per hermaphrodite parent worm is similar in *smcl-1(0)* mutants and wild-type controls (Fig 4B). Loss of condensin I function disrupts meiotic chromosome segregation, readily scored as an increase in the frequency of males (XO) produced by an XX hermaphrodite due to X-chromosome nondisjunction and production of gametes with no X chromosome. The *smcl-1(0)* mutant showed no such increase in the incidence of males compared to wild type (Fig 4C). Finally, we assessed hermaphrodite-specific lethality, a phenotype that results when condensin I^DC^ fails to downregulate expression of genes along hermaphrodite X chromosomes. To compare hermaphrodite to male viability, we mated XX hermaphrodites to XO males, which produces ~50% X0 male and 50% XX hermaphrodite progeny. Again, the *smcl-1(0)* worms did not show any change from the wild-type controls, and exhibited a similar 1:1 ratio of viable male and hermaphrodite progeny (Fig 4D). Consistently, we observed no obvious mis-localization of condensin complex subunits or changes to chromosome morphology in the *smcl-1* null mutant (S4 Fig). Thus, deletion of the *smcl-1* gene does not result in condensin phenotypes associated with condensin loss-of-function, suggesting that SMCL-1 is not required to promote the condensin functions that we examined.

Because we predicted that SMCL-1 is a regulator of condensin complexes, we reasoned that the loss of SMCL-1 function might have subtle effects in an otherwise wild-type animal but more pronounced effects when combined with a condensin subunit mutation. We therefore generated worms carrying both the *smcl-1(0)* mutation and a partial loss-of-function allele of *dpy-28*. The *dpy-28* gene encodes a CAPD-2 homolog shared between condensins I and I^DC^, and is represented by a temperature-sensitive hypomorphic allele (*dpy-28(y1)*, referred to as *dpy-28(ts)* here) that facilitates analysis of enhancement or suppression in double mutants [50, 60, 61]. At restrictive temperature, the *dpy-28(ts)* allele causes decreased embryo production (brood size) and hermaphrodite-specific embryonic or larval lethality. At semi-permissive temperature, *dpy-28(ts)* worms have somewhat larger brood sizes and some of these progeny escape early developmental defects and grow to the adult stage. Thus we counted the brood size and the number of progeny per worm that survive to adulthood to quantify the severity of the mutant phenotype.

At the semi-permissive temperature, the average number of viable adult progeny per worm in the *dpy-28(ts)* mutant was 8, but survival was increased to 25 in the *dpy-28(ts); smcl-1(0)* double mutant (Fig 4E, p<0.001). While 25 viable progeny per worm is not near the wild-type level of ~375 per worm (see Fig 4B), this suppression of *dpy-28(ts)* by the loss of *smcl-1* is statistically significant and supports the possibility that SMCL-1 protein may restrict condensin function. Similarly, when we scored brood size we found that while the average number of embryos produced by a *dpy-28(ts)* worm is 55, that number increased to 112 in the *dpy-28(ts); smcl-1(0)* double mutant (Fig 4F, p<0.01). For both phenotypes, we simultaneously assayed *dpy-28(ts); smcl-1(0); map*::*smcl-1* worms in which transgene-encoded wild-type SMCL-1 is present, and observed significant reversal of the adult viability suppression but not the brood size suppression (Fig 4E and 4F). A possible explanation is that the *map*::*smcl-1* rescue transgene may be expressed at lower than endogenous levels in the germline. Together, the mild phenotypic suppression of a hypomorphic *dpy-28* mutant by an *smcl-1* null mutant is consistent with a model that the SMCL-1 protein is a negative regulator whose removal allows improved function of condensin I and/or condensin I^DC^.

To test whether SMCL-1 might also regulate condensin II, we created double mutant between *smcl-1(0)* and the only other available temperature-sensitive condensin mutant, the condensin II allele *hcp-6(mr17)* [16, 62, 63]. We did not detect suppression of the *hcp-6(mr17)* low brood size or embryonic lethality phenotypes by *smcl-1(0)* at the temperatures we tested (S5A Fig). Therefore, it remains unclear whether SMCL-1 regulates condensin II.

### Overexpression of SMCL-1 causes chromosome segregation defects and disrupts condensin I^DC^ localization to X chromosomes

**Fig 5:**
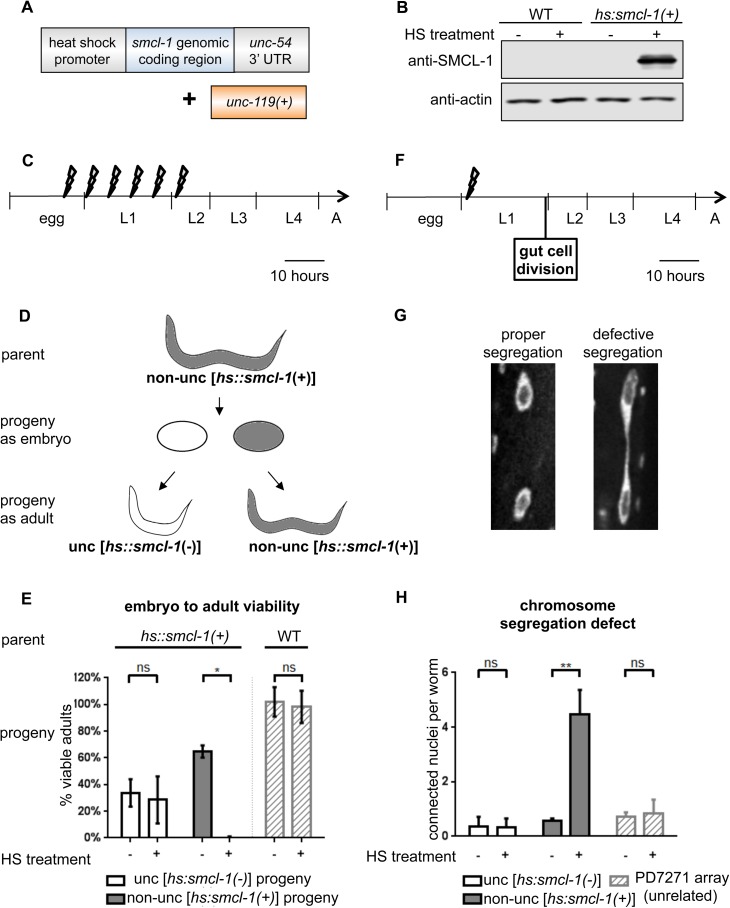
SMCL-1 overexpression leads to lethality and chromosome segregation defects. (A) The conditional overexpression transgene. The *smcl-1* genomic sequence is driven by the *hsp-16*.*48* promoter and contains the *unc-54* 3’UTR. The transgene was co-injected with *unc-119(+)* as the positive selection marker into *unc-119(ed9)* mutant worms. Worms containing multicopy transgene arrays with both the overexpression transgene and positive selection marker are designated *hs*:*smcl-1(+)*. (B) Western blot of standard lysate showing increase in SMCL-1 protein level after heat shock in *hs*:*smcl-1(+)* worms, compared to an actin control. (C) Heat shock regimen for data in (E). Bolt symbols represent heat-shock pulses administered from late embryo to early L2 larval stage. (D) Cartoon explaining segregation of the extrachromosomal transgene array. Parents that are *hs*:*smcl-1(+)* give rise to embryos without the overexpression array indicated *hs*:*smcl-1(-)*, and with the overexpression array indicated *hs*:*smcl-1(+)* and shaded gray; upon hatching, the progeny are identified by their uncoordinated and non-uncoordinated movements, respectively. Mosaics are also produced (not depicted). (E) Viability of progeny from *hs*:*smcl-1(+)* or wild-type (WT) parent worms after no heat shock (-, bottom) or after heat-shock pulses (+, bottom). For *hs*:*smcl-1(+)* parents, y-axis represents percent of embryos that develop into uncoordinated *hs*:*smcl-1(-)* adults without the transgene (white bars) or into non-uncoordinated *hs*:*smcl-1(+)* adults with the transgene (gray bars). Note no transgene-expressing progeny survive heat shock-induced *smcl-1* overexpression. For wild-type parents, y-axis represents percent of embryos that develop to adults (striped bars). (F) Heat shock regimen for data in (H). Bolt symbol represents a single 2-hour heat-shock pulse administered at early L1 larval stage to monitor effects of *smcl-1* overexpression on nuclear divisions in the gut that occur at late L1 larval stage. (G) Adult gut cells with DNA stained by DAPI and imaged by fluorescence microscopy. Connected chromatin between nuclei (right) indicates a prior chromosome segregation defect. (H) Chromosome segregation defect in the gut. L1 progeny of transgenic hermaphrodites were subjected to heat shock (+, bottom) or no heat shock (-, bottom) Some L1s were uncoordinated *hs*:*smcl-1(-)* L1 larvae without the transgene (white bars); others were non-uncoordinated *hs*:*smcl-1(+)* with the *smcl-1* overexpression transgene (gray bars). The number of connected nuclei per worm, which indicates defective chromosome segregation, was scored in >40 worms of each genotype. PD7271 is a control strain that contains an unrelated extrachromosomal array. HS = heat shock. ** denotes p ≤ 0.01; * denotes p ≤0.05; ns = not significant by a non-parametric Mann-Whitney test. Bars represent 95% confidence intervals.

If SMCL-1 is a regulator that restricts condensin function, then excess SMCL-1 protein is predicted to cause condensin loss-of-function phenotypes. To test this, we constructed a conditional transgene, *hs*::*smcl-1(+)*, that ubiquitously overexpresses SMCL-1 upon heat-shock induction (Fig 5A). We injected this transgene along with the *uncoordinated-119 (unc-119)* gene as a co-transformation marker into *unc-119(ed9)* mutant animals, which permitted the identification of worms carrying the extrachromosomal multicopy transgene array by rescue of the uncoordinated movement (“unc” phenotype) to normal non-uncoordinated movement (“non-unc” phenotype). While levels of SMCL-1 protein are low in wild-type adults and not detected in standard lysates, animals carrying the transgene showed high levels of SMCL-1 protein after heat shock induction (Fig 5B). Immunofluorescence revealed SMCL-1 overexpression throughout tissues and stages, but not in the germline and early embryos (S6A Fig), consistent with germline silencing typically observed for multicopy transgene arrays in *C*. *elegans*.

The mitotic and dosage compensation roles of *C*. *elegans* condensins are required for viability, so we scored survival after heat shock in animals with and without the heat shock-inducible *smcl-1* transgene array. Multiple heat-shock pulses were administered from late embryogenesis through larval stage L2 (Fig 5C), and we scored viability in adults that do not contain the transgene (unc phenotype) and those that carry the transgene (non-unc phenotype, Fig 5D). In control wild-type animals with no transgene, almost all embryos (98%) survive to adulthood after heat shock (Fig 5E, right). In contrast, animals carrying the *hs*::*smcl-1* transgene array did not survive the heat shock. To interpret these experiments, it is important to note that our heat shock-inducible *smcl-1* transgene was created as a multicopy extrachromosomal array in the *unc-119(ed9)* background, and it is not stably transmitted through cell division (in contrast to the integrated, *map*::*smcl-1* transgene). Thus, non-unc worms carrying the inducible *smcl-1* overexpression array [*hs*::*smcl-1(*+) *unc-119(+)*] can give rise to unc progeny that do not carry the array [*hs*::*smcl-1(-) unc-119(-)*] and non-unc progeny that carry the array [*hs*::*smcl-1(*+) *unc-119(+)*] (Fig 5D). Mosaic progeny with a mixture of cells with and without the transgene array are also produced (not depicted) and will be non-unc if sufficient cells carry the array. When embryos of transgenic worms were not exposed to heat shock, almost all survived to adulthood, with ~34% of embryos becoming unc adults without transgene (Fig 5E, HS-, white bar), and ~65% of embryos becoming non-unc adults with transgene (Fig 5E, HS–, gray bar). In contrast, when embryos of transgenic worms were subjected to heat shock pulses, all embryos hatched into L1 larvae and ~36% developed into unc adults without transgene (Fig 5E, HS+, white bar), while the remainder did not survive (Fig 5E, HS+, middle). The complete absence of transgene-expressing survivors indicates that prolonged overexpression of SMCL-1 during late embryogenesis and larval stages causes larval lethality.

To test if the SMCL-1 overexpression lethality phenotype was due to disrupting condensin function, we reduced the heat-shock regimen to a single non-lethal induction and assayed its effect on two known condensin functions: chromosome segregation (condensins I & II) and dosage compensation (condensin I^DC^). To assay for chromosome segregation defects, we performed heat shock at early L1 larval stage (Fig 5F), so excess SMCL-1 would be present when intestinal nuclei divide at the end of the L1 stage. We waited until adult stage to look for the unresolved DNA “bridges” between nuclei that indicate chromosome segregation defects (Fig 5G), because intestinal nuclei undergo rounds of DNA replication without division (endoreduplication) during the larval stages, so that by the adult stage nuclei are large and easier to visualize. Wild-type adults have low levels of unresolved DNA [16], as did *hs*::*smcl-1(-)* adults with and without heat shock (average of 0.4 and 0.3 connected nuclei per worm, respectively; Fig 5H, left). By contrast, in *hs*::*smcl-1(+)* worms, we observed an increase in connected nuclei per adult worm from 0.6 in the no-heat-shock control to 4.3 in the heat shock-induced SMCL-1 overexpression (Fig 5H, middle, p<.001). This represents a substantial defect since only 10-14 cells undergo this division, and if all had failed chromosome segregation, 10-14 connected nuclei would have been observed. To rule out the possibility that any extrachromosomal array could predispose the nucleus to more segregation defects upon heat shock, we also assayed worms containing an unrelated extrachromosomal array and did not observe an increase in connected nuclei (Fig 5H, right). Thus, overexpression of SMCL-1 causes a chromosome segregation defect in intestinal nuclei similar to that observed upon mutation or depletion of condensin I or II subunits [16].

**Fig 6:**
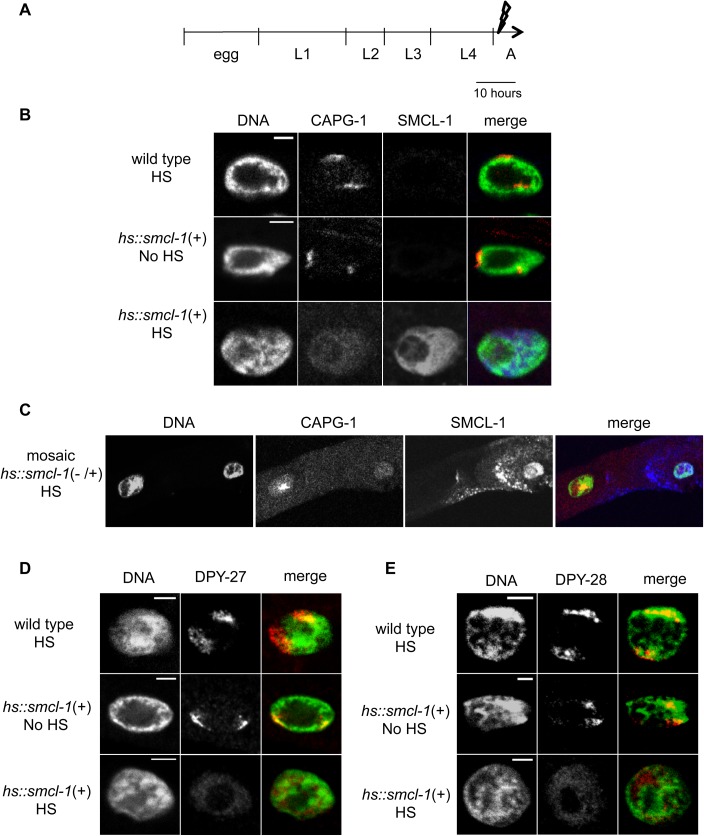
SMCL-1 overexpression in the gut disrupts condensin I^DC^ localization on the X chromosomes. (A) Heat shock regimen for data in (B-E). Bolt represents the single heat-shock pulse given to young adult hermaphrodites from the wild-type or inducible *hs*:*smcl-1(+)* transgenic strain. (B-E) Adult hermaphrodite gut tissue of the indicated strain and treatment was stained with DAPI to image DNA (green in merge) and immuno-stained with antibody against CAPG-1(B-C), DPY-27 (D) and DPY-28 (E), (red in merges). Antibody against SMCL-1 was also included in (B and C), showing overexpression upon heat shock (blue in merge). The foci of staining created by condensin I^DC^ subunit association with the X chromosomes are lost when SMCL-1 is overexpressed. (C) A mosaic animal in which anti-SMCL-1 staining indicates that one cell lacks the transgene (left) and shows foci of anti-CAPG-1, while a neighboring cells has the *smcl-1* overexpression transgene (right) and CAPG-1 staining is weak and not localized to foci (also see S6C and S6D Fig). HS = heat shock.

To study the effect of SMCL-1 overexpression on dosage compensation, we examined condensin I^DC^ localization to X chromosomes by immunofluorescence microscopy. Adult hermaphrodites carrying the overexpression transgene array were subjected to heat shock to induce SMCL-1 overexpression (Fig 6A). After four hours worms were co-stained with a DNA dye and antibodies against SMCL-1 and against the condensin I/I^DC^ subunit CAPG-1, and the large intestinal cell nuclei were examined. Heat-shocked wild-type animals and non-heat-shocked transgenic animals were used as controls. In control animals, no SMCL-1 signal was detected, consistent with our previous inability to detect SMCL-1 with our antibody or transgene in adult somatic nuclei; however, CAPG-1 is clearly visible in two sub-nuclear regions known to represent the two X chromosomes (Fig 6B top and middle) [16]. In contrast, when the *hs*::*smcl-1(+)* transgenic strain was subjected to heat shock, a strong SMCL-1 signal was observed throughout the nucleus and CAPG-1 appeared diffuse and no longer localized to distinct domains within the nucleus (Fig 6B, bottom). Providing an internal control, mosaic transgenic worms that were mixtures of *hs*::*smcl-1(+)* and *hs*::*smcl-1(-)* cells showed foci of CAPG-1 in nuclei with undetectable SMCL-1 signal, but not in adjacent nuclei with strong SMCL-1 signal (Fig 6C). We examined two other condensin I^DC^ subunits, DPY-27 and DPY-28, and found that heat shock of the *hs*::*smcl-1 (+)* transgenic strain similarly disrupted their association with X chromosomes (Fig 6D and 6E). It is not clear whether disrupted condensin I^DC^ localization is due in part to protein degradation: quantitative Western blot analysis revealed no significant difference in protein levels of DPY-27, DPY-28, and CAPG-1 between heat shock-induced *hs*::*smcl-1(+)* and wild-type adults (S6B Fig); however, quantification of immunofluorescence signals showed that levels of CAPG-1 are somewhat lower upon SMCL-1 overexpression (S6C and S6D Fig). Thus, overexpression of SMCL-1 prevents the normal localization of condensin I^DC^ complex proteins to hermaphrodite X chromosomes.

Taken together, SMCL-1 overexpression leads to a set of phenotypes similar to those observed upon loss of condensin I^DC^ and condensin I or condensin II function. The observed chromosome mis-segregation (Fig 5G and 5H) and condensin I^DC^ mis-localization (Fig 6) are consistent with the lethality (Fig 5E) observed after SMCL-1 overexpression. The finding that transgene-induced overexpression of SMCL-1 in intestinal cells disrupts mitosis and X-chromosome localization of condensin I^DC^ suggests that that the normal role of SMCL-1 might be negative regulation of condensin complexes.

### Evolution of SMCL-1

**Fig 7:**
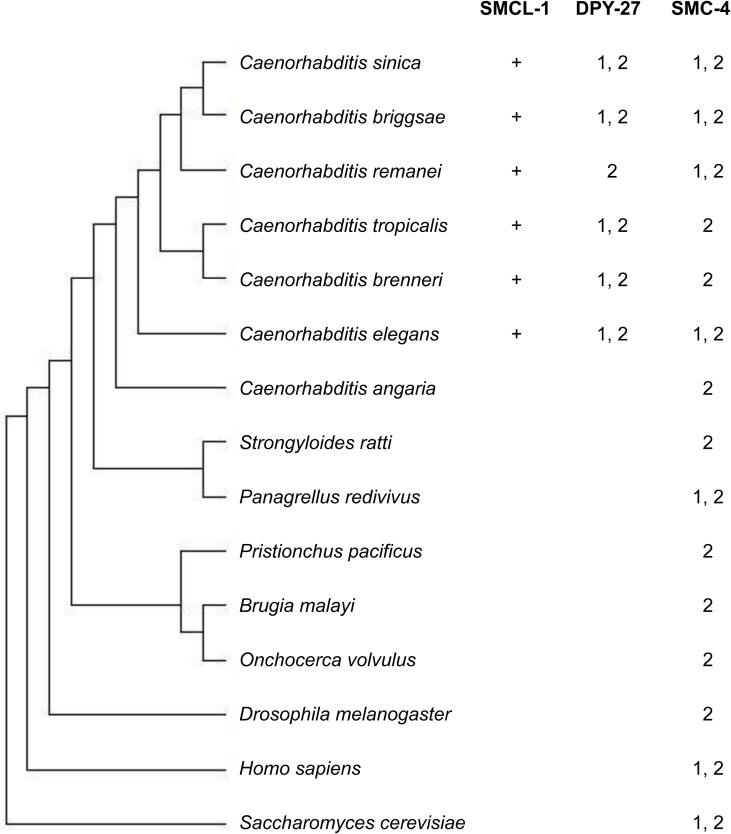
Presence of predicted orthologs of SMCL-1, DPY-27 (I^DC^), and SMC-4 (I & II) in various species. Phylogenetic tree built from all available *Caenorhabditis* species with sequenced and well-assembled genomes, other selected nematode species, and other selected model organisms. “*+”* symbol denotes the presence of SMCL-1-like protein based on similarity in a BLAST search and the additional criteria of short length, imperfect signature motif, and a Walker B motif lacking the catalytic glutamate (see Methods and S3 Fig). “1” denotes orthologs detected using a high-confidence Ensemble-COMPARA method and “2” denotes orthologs detected using BLAST-neighbor-joining tree methods.

We wondered whether SMCL-1 was widely conserved among organisms, or perhaps more recently evolved in lineages that acquired a third condensin complex specialized for dosage compensation. To address this question, we used a combination of BLAST (Basic Local Alignment Search Tool) [64], Ensembl-COMPARA [65] and Clustal Omega methods [66] to search available genomes of *Caenorhabditis* species, several other nematode species, and *H*. *sapiens*, *D*. *melanogaster*, and *S*. *cerevisiae*, for the presence of proteins resembling SMCL-1, SMC-4, and DPY-27 (Methods, Fig 7, S3 Fig).

Conventional orthology prediction programs such as Ensembl-COMPARA and Treefam were not able to identify orthologs of SMCL-1. To search for SMCL-1-like proteins in the indicated species, we initiated a BLASTP search using *C*. *elegans* SMCL-1 and then determined which of the resulting proteins had the SMCL-1 features of an imperfect signature motif, followed by 18 amino acids and a Walker B motif lacking the catalytic glutamate (see Fig 3D). We identified only 10 other proteins that fit these criteria, all in *Caenorhabditis* species, including *C*. *briggsae*, *C*. *remanei*, *C*. *brenneri*, *C*. *sinica* and *C*. *tropicalis*. (Fig 7, S3 Fig). When we further examined these proteins, we found they shared other sequence similarities with SMCL-1, including 1) shorter length than typical SMC proteins, 2) presence of a Walker A motif, 3) lack of SMC hinge domain, 4) lack of predicted coiled-coils (S3 Fig). We did not find proteins resembling SMCL-1 in *C*. *angaria*, or in more distantly related nematodes like *P*. *pacificus*, or in organisms like *S*. *cerevisiae*, *D*. *melanogaster*, or *H*. *sapiens*.

Interestingly, the set of examined species that encode an SMCL-1 protein coincides with species that encode both SMC-4 and DPY-27-like proteins, which we identified by two methods, Ensembl-COMPARA and BLAST-Clustal Omega (indicated as 1 and 2, respectively in Fig 7, see Methods). This suggests that the biological role of SMCL-1 may be related to the presence of condensin I^DC^. We speculate that SMCL-1 may have arisen to help organisms cope with the deployment of shared subunits among three condensin complexes.

## Discussion

### Why is regulation of condensin important?

Differential regulation of condensin activity allows condensins to arrange chromosomes into the different topologies required for processes like mitosis/meiosis, gene expression, recombination, and DNA repair. The chromosomal binding sites and activity of condensins dramatically change during different phases of the cell cycle, developmental stages and cell types, and in response to environmental triggers like starvation or DNA damage. In *C*. *elegans* and other organisms, particular DNA sequence motifs, recruitment proteins, and post-translational modifications have been shown to promote the binding and activity of the different condensin complexes. Here we identified the novel SMC-like protein SMCL-1 through its physical interaction with condensin SMC subunits, and showed that it can influence the localization and function of *C*. *elegans* condensin complexes.

Biochemical and phenotypic data suggest that SMCL-1 is not a canonical member of a condensin complex, but may regulate one or more condensin complexes. SMCL-1 may negatively regulate the *C*. *elegans* condensin that specializes in X-chromosome dosage compensation: SMCL-1 binds to the condensin I^DC^-specific subunit DPY-27, the loss of *smcl-1* function partially rescues lethality of a *dpy-28* (condensin I/I^DC^) mutant, and overexpression of SMCL-1 causes lethality and displaces condensin I^DC^ from X chromosomes. Future studies can address whether *smcl-1* shows genetic interactions with non-condensin dosage compensation factors like *dpy-21*, *sdc-2*, and *sdc-3*; preliminary results showed that loss of *smcl-1* did not suppress lethality of a *dpy-21* mutant (S5 Fig) [67]. SMCL-1 may also regulate the mitotic/meiotic condensin I complex: SMCL-1 binds to SMC-4 (condensins I & II) and weakly to CAP I subunits (condensin I & I^DC^), the loss of *smcl-1* partially suppresses the low brood size of a *dpy-28* mutant (condensin I/I^DC^), and overexpression of SMCL-1 causes chromosome segregation defects characteristic of condensin I or II mutants. We cannot rule out that SMCL-1 influences condensin II since SMCL-1 interacts with SMC-4 (condensins I & II) and its overexpression leads to chromosome segregation defects and lethality, but we note that SMCL-1 did not co-purify with condensin II CAP subunits and under tested conditions the loss of *smcl-1* did not suppress lethality of a condensin II mutant. Future studies will be required to further define which of the three complexes and their multiple activities are modulated by SMCL-1.

What could negative regulation of *C*. *elegans* condensins achieve? In other systems such as *Xenopus* egg extracts, mitotic chromosomes are compacted laterally by condensin I and longitudinally by condensin II, and changing the ratio of condensin I to II alters chromosome shape [24]. Accordingly, loss of negative regulation of condensin II in *Drosophila* and vertebrates causes over-condensation and axial shortening of interphase chromosomes [32, 34, 35, 68]. Because condensins I and II share the same SMC subunits but use different CAP subunits, the ratio of condensin I to II is determined by the relative abundance of class I or class II CAP subunits. In *C*. *elegans*, the evolutionary emergence of an additional condensin I^DC^ complex that uses the same class I CAP subunits as condensin I may have interfered with typical modes of regulation, and required new modes of regulation. We showed that SMCL-1 binds to the SMC subunits of all three condensin complexes, while binding very little class I and no class II CAP subunits. We speculate that SMCL-1 could sequester away SMC subunits, allowing independent regulation of CAP subunits and facilitating the exchange and distribution of shared subunits among three partially overlapping complexes. Such regulation might help generate the appropriate ratio of each condensin complex in a cell at a particular stage of the cell cycle or development. We note that SMCL-1 protein showed most expression in the germline, where chromosomes undergo drastic shape changes. In support of the idea that regulation by SMCL-1 arose to help organisms adapt to the challenges of having yet a third condensin complex with shared subunits, SMCL-1 is present in a set of *Caenorhabditis* lineages that also have the condensin I^DC^-specific subunit DPY-27 (Fig 7 and see below).

A small but growing list of negative regulators of condensins has been identified. Like previously identified negative regulators, loss of SMCL-1 partially rescued defects of a hypomorphic condensin mutant, and overexpression of SMCL-1 caused the lethality, chromosome segregation, and condensin mis-localization defects associated with condensin loss-of-function mutants. Some *Drosophila* proteins negatively regulate condensin proteins via degradation [34, 35], and it is not clear whether SMCL-1 may reduce condensin subunit levels (S6). Unlike previously identified negative regulators of condensin II [34, 35, 68], loss of SMCL-1 did not cause obvious chromosome over-condensation (S4 Fig). In fact, phenotypes from loss of *smcl-1* were only obvious when condensin function was partially compromised (Fig 4). This finding suggests that SMCL-1 may be a subtle modulator, redundant, or more critical in response to an as-yet identified environmental trigger.

### How does SMCL-1 regulate condensins?

We propose two classes of models to explain how SMCL-1 could modulate condensin function. The first model proposes that when SMCL-1 binds to condensin SMC proteins, it alters the ATPase cycle required for condensin function. Typical SMC proteins fold at a central hinge, bringing together coiled regions and joining the N- and C- termini into an ATPase head domain. SMC heterodimers contact at the hinge, and when ATP is bound they also contact at the head domains, which undergo cycles of ATP binding, head engagement, and ATP hydrolysis. Because SMCL-1 lacks a hinge domain required for hinge-hinge contact and lacks conserved amino acids in the signature motif required for canonical head-head engagement, it must bind to condensin SMC proteins in a manner different from typical SMC-SMC interactions. One possibility is that SMCL-1 forms a head domain that can bind to a condensin SMC head domain, but prevents ATP hydrolysis because SMCL-1 lacks the conserved catalytic amino acid in the Walker B motif. ATPase activity requires association of two functional head domains, and it has been shown that SMC proteins engineered to lack the catalytic amino acid dominantly inhibit the ATPase activity of wild-type SMC proteins [52, 57]. It is also known that intermolecular interactions between two head domains of SMC proteins can occur even when they have a mutation that prevents hinge-hinge interaction and a mutation that prevents ATP hydrolysis but not binding [11]. While the notable change in its Walker B motif suggests that SMCL-1 may be a “poison head domain,” it could alternatively disrupt the ATPase cycle and/or SMC complex ring structure by physically interacting with condensin SMCs in a way that distorts the SMC protein or occludes its normal protein-protein interactions.

A predicted consequence of interfering with condensin’s ATPase cycle is disrupting aspects of its chromosome binding [10, 11, 52, 55]). We observed that overexpression of SMCL-1 disrupts condensin I^DC^ localization to X chromosomes. In adults, condensin I^DC^ is present on X chromosomes, and upon SMCL-1 overexpression appeared to lose its X association. It will be of interest to learn whether SMCL-1 intersects with previously identified loading factors and post-translational modifiers that regulate *C*. *elegans* condensin localization.

A second model is that SMCL-1 regulates condensins by binding and sequestering core condensin SMC subunits to prevent their interaction with CAP subunits, preventing formation of functional holocomplexes. This model is suggested by our finding that SMCL-1 purification recovered many more peptides corresponding to each of the three SMC subunits (DPY-27, SMC-4, and MIX-1) than to the CAP I subunits, and did not recover any CAP II subunits. The SMCL-1-SMC protein interaction interface, which remains to be determined, could prevent interactions between SMC proteins and the kleisin CAP subunit. A speculative possibility is that SMCL-1 regulates the availability of SMC proteins free for interaction with CAP subunits, to help govern the ratio of the three condensin complexes. Future experiments will be required to develop in *vitro* ATPase assays with purified *C*. *elegans* proteins and other assays to test the biochemical predictions of these models, to determine if SMCL-1 works by one or a combination of these mechanisms.

### Evolution of SMCL-1

SMCs are ancient chromosomal proteins found in all domains of life. SMC family proteins typically partner with a second SMC protein and accessory proteins. Over the course of evolution, the duplication and divergence of SMC complex members has led to the elaboration of multiple SMC complexes with diverse roles in chromosome dynamics. For example, most eukaryotes have SMC1/3 cohesin, SMC2/4 condensin, and SMC5/6 DNA repair complexes [3]. Additional duplication and divergence events have led to specialized versions of these complexes. Specialized condensins arose from the duplication and divergence of CAP subunits to form condensins I and II, and the duplication and divergence of SMC-4 to create *C*. *elegans* DPY-27 and to form condensin I^DC^.

Here we uncovered a new variation on this theme. SMCL-1 also appears to have arisen by duplication and divergence of an SMC gene, but in contrast to previous examples it appears to have acquired a role in modulating the activity of *C*. *elegans* condensin complexes. Homology searches indicate that a subset of *Caenorhabditis* species have proteins like SMCL-1. Interestingly, species with SMCL-1-like proteins are the ones that also harbor homologs of both SMC-4 (condensin I and II) and the condensin I^DC^-specific SMC4 class protein DPY-27. Although higher resolution phylogenetic studies are needed to determine co-evolution, the apparent co-emergence of SMCL-1 and DPY-27 during evolution leads us to speculate that SMCL-1 arose to meet the regulatory challenges imposed by the introduction of a third condensin complex. We did not find SMCL-1-like proteins outside of a subset of *Caenorhabditis* species, but it remains possible that some other species independently evolved similarly duplicated and diverged partial SMC proteins. In fact, another example has been reported of a truncated SMC protein with non-canonical functions. The vertebrate Structural Maintenance of Chromosomes Hinge Domain 1 protein (SMCHD1) resembles only the hinge domain of SMC proteins and functions in epigenetic gene regulation of X chromosomes and imprinted loci [69]. It is intriguing to note that both examples of truncated SMCs are implicated in X-chromosome gene dosage regulation, a rapidly evolving process whose mechanism differs among organisms but commonly involves alterations to chromatin structure.

Our work, along with prior findings, illustrates how genes encoding SMC family proteins and their partners have served as the raw material for evolutionary innovation. The duplication and divergence of modules of these chromosomal machines has allowed the creation of new protein ensembles with new functions, or as shown here has created a regulator of such complexes. The discovery of non-canonical SMC proteins like SMCHD1 and SMCL-1 suggests that other seemingly odd SMC-like homologs in genomes may warrant investigation as potential new players in chromosome maintenance.

## Methods

### *C*. *elegans* strains and growth

Worms were maintained at 20°C unless otherwise noted, on NG plates, and fed *E*. *coli* OP50 with the exception of worms used for purification, which were fed HB101.

The following worm strains were used or generated:

EG4322 *ttTi5605 II; unc-119(ed9) III*

KIR3 *stnSi1[kle-2 promoter*:*map*::*kle-2(+)*:*kle-2 3’UTR cb-unc-119(+)] II; kle-2(ok1151) III*

KIR4 *stnSi2[dpy-27 promoter*:*map*::*dpy-27(+)*:*dpy-27 3’UTR cb-unc-119(+)] II; dpy-27(tm3326) III*

KIR5 *stnSi3[dpy-26 promoter*:*dpy-26(+)*::*map*:*dpy-26 3’UTR cb-unc-119(+)] II; dpy-26(tm3432) IV*

KIR6 *ttTi11701 X; unc-119(ed9) III*

KIR7 *smcl-1(stn1) X* = *smcl-1[stn1*::*cb-unc-119(+)] X*

KIR8 *stnSi4[smcl-1 promoter*:*map*::*smcl-1(+)*:*smcl-1 3’UTR cb-unc-119(+)] II; smcl-1(stn1) X*

KIR9 *stnEx1[hsp-16*.*48 promoter*:*smcl-1(+)*:*unc-54 3’UTR cb-unc-119(+)]; unc-119(ed9) III*

TY148 *dpy-28(y1) III*

KIR10 *dpy-28(y1) III; smcl-1(stn1) X*

KIR11 *dpy-28(y1) III; smcl-1(stn1) X; stnSi4*

### Generating fusion constructs and single-copy transgenic strains

Worms containing MAP-tagged condensin subunits and SMCL-1 were generated according to [46]. Briefly, plasmids containing the endogenous 5’ element (1 kb upstream of start), MAP fusion transgene and 3’UTR (0.5 kb downstream of start) were constructed from MosSCI destination vector pCFJ150 (Addgene, Cambridge, MA) [70] using the Multisite Gateway Three-fragment Construction Kit (Invitrogen, Carlsbad, CA). Plasmids were injected into worm germlines to generate single-copy transgenes using the direct insertion MosSCI technique [70]. Transgenic worms were outcrossed to wild-type worms before crossing into null mutants.

### Generating *smcl-1* deletion allele

The *smcl-1* deletion allele was generated using the MosDEL technique [58] with ttTi11701, an allele that harbors a Mos1 transposon insertion 335 bp upstream of the *smcl-1* ORF, generated by the NemaGENETAG consortium [71]. In the presence of Mos transposase, the transposon is cut to generate site-specific double-strand break that allows for repair through recombination. To generate the recombination plasmid, three PCR fragments: 3kb upstream of Mos transposon insertion site, *cb-unc119* and 2 kb downstream of *smcl-1* ORF, were stitched together using PCR fusion [72] and recombined into pDONR P4-P1R (attB4 attB1r primers used) using Gateway BP Clonase II (Invitrogen). The resulting plasmid was then co-injected with transposase plasmid and mCherry according to [58] into ttTi11701; *unc-119(ed9)*. The deletion strain was outcrossed to wild-type worms four times.

### Generating heat-shock inducible SMCL-1 transgenic strain

PCR fusion was used to generate a fragment containing the *hsp-16*.*48* promoter, *smcl-1* genomic coding region (start codon to stop codon, including introns) and *unc-54* 3’UTR. The fragment was transformed into pDONR P4-P1r using BP Clonase II to generate the *hs*::*smcl-1* overexpression plasmid. For the co-injection marker, *cb-unc-119* was PCR amplified and transformed into pDONR P2r-P3 also with BP Clonase II to generate the co-injection plasmid. The two plasmids were mixed and injected at 70 ng/uL each into 35 *unc-119(ed9)* P0 worms. Four independent stable transgenic lines were obtained from ~100 F1 transformants.

### Phenotype assays

#### Viable adult progeny (Fig 4B)

L4 hermaphrodites were picked to individual plates and transferred to new plates daily for egg laying. Total progeny that grew into L4 or adult per hermaphrodite were counted.

#### Viable adult progeny and brood size for strains containing *dpy-28(y1)* (Fig 4E and 4F)

L4 hermaphrodites were picked to individual plates and grown to adults at 15°C. Gravid adults were then picked to new plates at 20°C for overnight egg-lay. Resulting progeny were grown at 20°C to L4, picked onto individual plates, and transferred to new plates every 12 hours. These embryos were counted to determine brood size (Fig 4F) and then number of L4/adult progeny per hermaphrodite was counted to determine viable progeny (Fig 4E).

#### High incidence of males (X nondisjunction) assay (Fig 4C)

L4 hermaphrodites were picked to individual plates and transferred daily for egg laying. Total male and hermaphrodite progeny were counted.

#### Hermaphrodite to male ratio (Fig 4D)

L4 hermaphrodites were mated to males, and transferred to new plates daily. The total number of male or hermaphrodite progeny per mated hermaphrodite was counted.

#### Embryo to adult viability in SMCL-1 overexpression strain (Fig 5E)

L4 worms were picked to individual plates, grown until gravid at 15°C, then picked to a new plate for a two-hour egg lay. Total embryos were counted. At 14 hours after egg-lay, heat shock pulses to induce SMCL-1 overexpression were conducted as follows: Worms on plates were heated to 30°C by placing in a 34°C incubator for 2 hours, then plates were moved back to a 15°C incubator for 4 hours. This was repeated six times (36 hours) and plates were subsequently maintained at 15°C. Viable adults with unc or non-unc phenotypes were counted, to determine percent embryo to adult viability (Fig 5E).

#### Chromosome segregation defect in SMCL-1 overexpression strain (Fig 5G and 5H)

Gravid adults were picked to individual plates for a 2-hour egg-lay at 15°C. Embryos were allowed to hatch for 19 hours and young L1 worms on plates were shifted to 30°C to induce SMCL-1 overexpression (using a 34°C incubator) for 2 hours. Worms were subsequently maintained at 15°C until early, non-gravid, adult stage. Whole young adults were picked onto slides, treated with Carnoy’s fixative, rehydrated in 1x M9 buffer, and mounted in ProLong antifade containing DAPI. The number of pairs of nuclei with visible DAPI-stained connections (Fig 5G) was scored per worm (Fig 5H).

#### Tests of statistical significance

All statistical significance of differences was determined by calculating p values using non-parametric Mann-Whitney test.

### Extract preparation, Western blots, and quantification of condensin subunit levels

#### Sonicated lysate (Fig 4A and purifications in Figs 1 and 2)

At 4°C, adult hermaphrodites were subjected to extensive dounce homogenization and sonication in lysis buffer (50 mM HEPES-KOH, pH7.6, 150 mM KCl, 1 mM EDTA, 0.5% NP-40, 10% glycerol) containing protease inhibitor cocktail (Roche). Supernatant was collected after centrifugation for 20 min. at 16,000g.

#### Standard small-scale lysate (Figs 5B and S6B)

100 young adults were picked into 1 mL cold M9 buffer, settled, and all but 10 uL removed. Samples were flash frozen in liquid nitrogen, thawed, and 30 uL of Western sample buffer added to achieve final concentration of 1X Laemmli buffer, 1X protease inhibitor cocktail (Roche Complete), 0.1 M dithiothreitol and 5% β-mercaptoethanol. Then 40 uL of glass beads (Sigma G8772) were added and vortexed 10 min., with short incubation in ethanol/ice bath every 10 sec. Samples were then heated to 95°C for 10 min., centrifuged, and supernatant collected.

**Western blots** were performed using standard procedures, with condensin antibodies described in [16], mouse anti-actin (Abcam 3280), or anti-SMCL-1 raised in rabbit against a C-terminal peptide.

#### Quantification of condensin subunit levels (S6B Fig)

In four replicates, young adult *hs*::*smcl-1(+)* or wild-type worms were heat shocked for 2 hours or not heat shocked, recovered for 4 hours, then standard lysate was prepared. Western blots were probed with primary rabbit anti-condensin or anti-SMCL-1 and mouse anti-actin (Abcam 3280), secondary anti-rabbit with IRDye 800CW and anti-mouse with IRDye680RD, then imaged using the Odyssey CLx Imaging System and Image Studio software, and signal densities were normalized to actin as per manufacturer’s protocol (Li-Cor).

### Tandem affinity purification of MAP-tagged proteins and MudPIT mass spectrometry

Tandem affinity purifications of MAP-tagged proteins were performed according to [46]. Purifications were performed from sonicated lysates of hermaphrodites from null mutant strains rescued by corresponding MAP-tagged protein, such that only MAP-tagged bait proteins were present. MudPIT mass spectrometry was performed as described in [16]. Two replicas were performed; only proteins recovered in both replicas and not present in a control purification from untagged wild-type strain were considered. Relative protein abundance was estimated by calculating a NSAF (normalized spectral abundance factor): the number of spectra identified was normalized to protein length, and divided by the total spectra in the sample [47].

### Microscopy and immunohistochemistry

Live imaging of MAP-tagged protein fluorescence was performed by mounting worms as described in protocol 1 of [73] and observing on a Leica (Deerfield, Il) TCS SPII confocal microscope. For heat shock induction of SMCL-1 and antibody staining (HS, Fig 6), young adults grown at 15°C were shifted to 30°C (by placing plates in a 34°C incubator) for 2 hours. Control plates were kept at 15°C (no HS, Fig 6). At 4 hours after the end of heat shock, worm dissection and antibody staining were performed as described in protocol #21 in [73]. All images (Figs 3A, 5G, 6B–6E, S1, S4 and S6) were captured at 63x using a Leica (Deerfield, IL) TCS SPII confocal microscope.

### Bioinformatics analysis of SMCL-1, SMC-4, and DPY-27-like proteins

The search for SMCL-1-like proteins was performed using two methods based on protein BLAST. The first method involved PHI-BLAST using the syntax G-G-x(20)-[AVILMFYW]-[AVILMFYW]-[AVILMFYW]-[AVILMFYW]-D-[RHKDSTNQCGPAVILMFYW], followed by three iterations of PSI-BLAST using proteins with SMCL-1-like imperfect signature motif and a Walker B motif lacking the catalytic glutamate, as seed in the second and third iterations. PHI-BLAST and PSI-BLAST are available on blast.ncbi.nlm.nih.gov [64]. In the second method, the list of SMCL-1 homologs pre-calculated by Wormbase using WU-BLAST 2.0 [Gish, W. (1996–2003) http://blast.wustl.edu] was manually searched for proteins with SMCL-1-like imperfect signature and Walker B motifs. Expect value threshold was set at 0.00001 for all BLAST searches.

The search for SMC-4 and DPY-27-like proteins was performed using two methods. The first method used was the Ensembl-COMPARA orthology prediction method with confidence set to high, against *C*. *elegans* SMC-4 or DPY-27 [65]. Pre-calculated Ensembl-COMPARA results for the examined species are available on parasite.wormbase.org/biomart [74]. The second method was performed by first generating a list of the top full-length homologs of each of the seven *C*. *elegans* SMC proteins, followed by building of a neighbor-joining tree containing these proteins using Clustal Omega [66]. The tree that was generated split into clades representing each of the SMC proteins. The absence of protein representing a specific species in the SMC-4 or DPY-27 clade was interpreted as the absence of SMC-4 or DPY-27-like protein in that species. The absence of SMC-4 or DPY-27-like protein was further confirmed by individually including all other full-length homologs of SMC-4 or DPY-27 in the tree to search for potential orthologs that might share less similarity with the *C*. *elegans* proteins. All searches were limited to test species listed in Fig 7.

## Supporting information

S1 Fig MAP-tagged condensin transgenes are functional. (A) Summary of phenotypes observed in condensin homozygous mutants from a heterozygous mother (m+z-) or homozygous mutants from a homozygous mutant mother (m-z-) with indicated genotypes, and phenotypic rescue with the corresponding *map*::*subunit* transgene. (B) Chromosomal localization of MAP-tagged condensin transgenes in embryos stained with DAPI to visualize DNA (red) and antibody against mVenus to visualize the transgene (green), which showed expected patterns. (C) Sonicated lysates from a wild-type strain (lane 1) and a *dpy-27(tm3326);map*::*dpy-27* strain (lane 4), each containing 150 ug total protein, analyzed by Western blot probed with antibody against DPY-27, showing that the *map*::*dpy-27* transgene expresses at levels similar to endogenous DPY-27 levels. Similar results were obtained for KLE-2 [46]; DPY-26 was not assayed due to lack of appropriate antibody. Other lanes are eluates from 1-step or tandem purification experiments; size marker in kilodaltons shown to left.
(PDF)

S2 Fig Transcript levels of *smcl-1* and condensin subunit genes. RNA levels in fragments per million reads (FPKM) are mean values compiled by Wormbase from multiple published RNA sequencing datasets (www.wormbase.org).
(PDF)

S3 Fig Comparison between *C*. *elegans* SMCL-1 and SMCL-1 like proteins defined in other species. A set of species was examined for SMCL-1 like proteins based on SMC homology, intact Walker A motif, and non-conserved signature motif and Walker B motifs (see Methods, Results, and Fig 7). The left column shows proteins identified this way as well as a canonical SMC and SMCL-1 for reference; other columns indicate relevant protein features. Conserved motif sequences are shown below the motif labels in the first row. *x* represents any amino acid; *h* represents any hydrophobic amino acid; *+* represents amino acid in agreement with consensus.
(PDF)

S4 Fig *smcl-1* deletion does not cause obvious mis-localization of condensin subunits or changes to chromosome morphology. (A) DNA stain (green) and antibody against DPY-27 (red) in oocytes showing diffuse nuclear DPY-27 staining in both wild-type and *smcl-1(0)*. Antibody against SMC-4 in *smcl-1(0)* embryo (bottom), showing typical localization to anaphase chromosomes [14]. (B) Comparison of chromosome morphology in wild-type and *smcl-1(0)* during meiotic pachytene and diakinesis (top), mitotic metaphase and anaphase in a 2-cell embryo (middle), and in early embryo interphase nuclei (bottom).
(PDF)

S5 Fig Phenotypes of *hcp-6(mr17); smcl-1(0)* and *dpy-21(e428); smcl-1(0)* double mutants. (A) Brood size and viability phenotypes of *hcp-6(mr17)*, a temperature-sensitive allele of a condensin II subunit, alone and in combination with the *smcl-1* null allele. Top row shows results for shift from 15°C to 21.5°C at larval L1 stage, then brood counted from 3 hour egg lay of young adult. Middle shows results for shift from 15°C to 25°C at young adult stage, then total brood counted. Both are averages of two biological replicas, with >10 hermaphrodites of each genotype per replica. Bottom row shows results for shift from 15°C to 21.5°C at young embryo stage, then scored for percent of embryos laid that survive to hatch into L1 larvae, averaged from three replicas with >70 embryos per replica. (B) Average number of viable adult progeny per worm over its lifetime measured for the *e428* allele of *dpy-21*, whose product is required for X dosage compensation but is not part of condensin I^DC^ [67], alone or in combination with the *smcl-1* null allele.
(PDF)

S6 Fig Analysis of SMCL-1 overexpression on condensin subunit protein levels. (A) Example of inducible *hs*:*smcl-1(+)* transgenic strain after heat shock, stained for DNA, CAPG-1, and SMCL-1, showing SMCL-1 overexpression is induced in the gut but not the germline. (B) Wild-type (white) or *hs*::*smcl-1(+)* (gray) young adults were subjected to 2 hours of heat shock (HS+, bottom) or no heat shock (HS-, bottom), recovered for 4 hours, then standard lysates prepared and analyzed on Western blots with antibodies against actin, the condensin subunits shown, and SMCL-1. Signals were quantified on an Odyssey CLx Imaging system and normalized using relative actin signal density. Mean values from 4 biological replicas are shown. No significant change in levels of each subunit was detected upon heat shock-induced SMCL-1 overexpression; p-values calculated by non-parametric Mann-Whitney test. Bars represent 95% confidence intervals. (C, D) Gut nuclei from the *hs*:*smcl-1(+)* mosaic strain after heat shock, stained for DNA (top), and immuno-stained for CAPG-1 (middle) and SMCL-1 (bottom). (C) Cell at right shows no SMCL-1 overexpression and CAPG-1 is localized in the nucleus with enrichment at a sub-nuclear focus (X chromosome). Cell at left shows SMCL-1 overexpression and lower levels of diffuse nuclear CAPG-1 staining and no X localization. Single confocal plane shown. (D) Stacked confocal images of another pair of nuclei (left) and quantification of pixel intensity (right) across a line (visible in CAPG-1 panel) drawn left to right, suggesting CAPG-1 is present but reduced in the SMCL-1 overexpressing nucleus.
(PDF)

S1 Table Proteins identified by tandem purification against MAP tag using *dpy-27(0); map*::*dpy-27* lysates. (XLSX)

S2 Table Proteins identified by tandem purification against MAP tag using *dpy-26(0); dpy-26*::*map* lysates. (XLSX)

S3 Table Proteins identified by tandem purification against MAP tag using *kle-2(0); map*::*kle-2* lysates. (XLSX)

S4 Table Proteins identified by tandem purification against MAP tag using untagged wild-type control lysates. (XLSX)

S5 Table Proteins identified by tandem purification against MAP tag using *smcl-1(0); map*::*smcl-1* lysates. (XLSX)

S6 Table Proteins identified by 1-step purification against MAP tag using *dpy-27(0); map*::*dpy-27* lysates. (XLSX)

S7 Table Proteins identified by 1-step purification against MAP tag using *dpy-26(0); dpy-26*::*map* lysates. (XLSX)

S8 Table Proteins identified by 1-step purification against MAP tag using *kle-2(0); map*::*kle-2* lysates. (XLSX)

S9 Table Proteins identified by 1-step purification against MAP tag using *smcl-1(0); map*::*smcl-1* lysates. (XLSX)

S10 Table Proteins identified by 1-step purification against MAP tag using untagged wild-type control lysates. (XLSX)
