# Supplementary material for: An SMC-like protein binds and regulates Caenorhabditis elegans condensins
Source: PLoS Genet. 2017 Mar 16;13(3):e1006614. doi: 10.1371/journal.pgen.1006614 (PMC5373644; doi:10.1371/journal.pgen.1006614)

**A**

| genotype | ***dpy-27(tm3326)*** | ***dpy-26(tm3432)*** | ***kle-2(ok1151)*** |
|---|---|---|---|
| ***m+z-* mutant** | dumpy | dumpy | germline-less, sterile, uncoordinated, protruding vulvae |
| ***m-z-* mutant** | dumpy; sterile | dumpy; sterile | n/a |
| ***m-z- mutant; map-tagged transgene*** | appear wild-type | appear wild-type | appear wild-type, but temperature-sensitive |

**B**

*dpy-27(tm3326); map::dpy-27*    *dpy-26(3432); dpy-26::map*    *kle-2(ok1151); map::kle-2*

anti-GFP

DNA

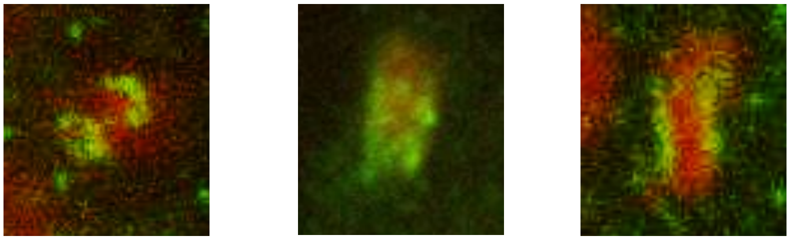

**C**

**WT extract**    ***dpy-27(tm3326); map::dpy-27* extract**

**1 2 3 4**

250

150

**anti-DPY-27**

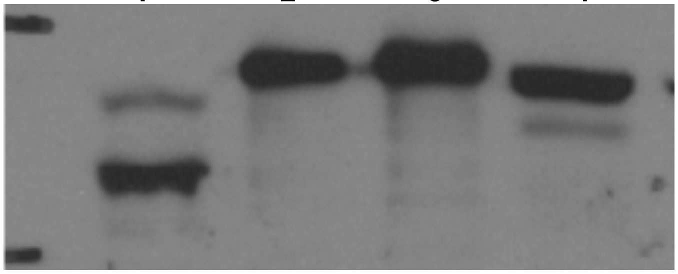

Supplement: S1 Fig — (A) Summary of phenotypes observed in condensin homozygous mutants from a heterozygous mother (m+z-) or homozygous mutants from a homozygous mutant mother (m-z-) with indicated genotypes, and phenotypic rescue with the corresponding map::subunit transgene. (B) Chromosomal localization of MAP-tagged condensin transgenes in embryos stained with DAPI to visualize DNA (red) and antibody against mVenus to visualize the transgene (green), which showed expected patterns. (C) Sonicated lysates from a wild-type strain (lane 1) and a dpy-27(tm3326);map::dpy-27 strain (lane 4), each containing 150 ug total protein, analyzed by Western blot probed with antibody against DPY-27, showing that the map::dpy-27 transgene expresses at levels similar to endogenous DPY-27 levels. Similar results were obtained for KLE-2 [46]; DPY-26 was not assayed due to lack of appropriate antibody. Other lanes are eluates from 1-step or tandem purification experiments; size marker in kilodaltons shown to left. (PDF) [file pgen.1006614.s001.pdf]
