# Supplementary figures and images for: An SMC-like protein binds and regulates Caenorhabditis elegans condensins
Source: PLoS Genet. 2017 Mar 16;13(3):e1006614. doi: 10.1371/journal.pgen.1006614 (PMC5373644; doi:10.1371/journal.pgen.1006614)

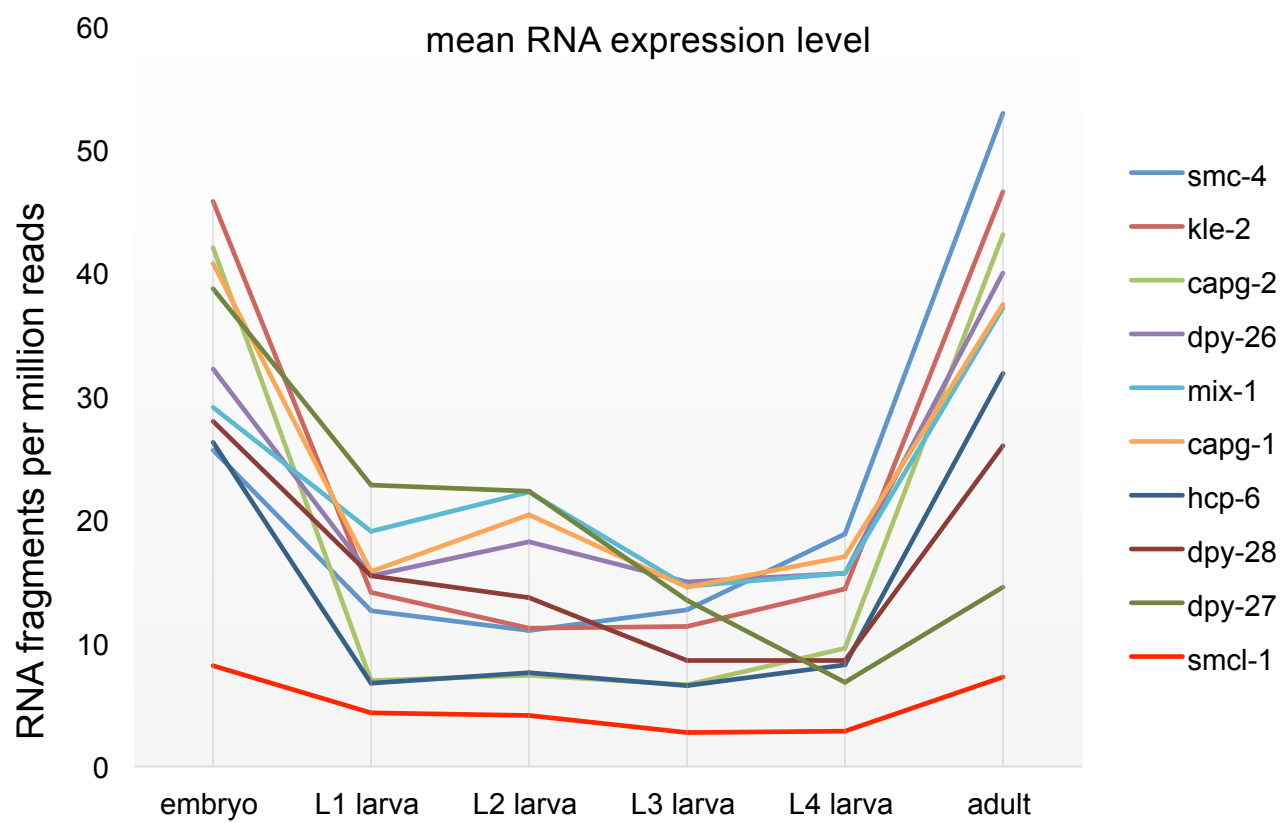

Supplement: S2 Fig — RNA levels in fragments per million reads (FPKM) are mean values compiled by Wormbase from multiple published RNA sequencing datasets (www.wormbase.org). (PDF) [file pgen.1006614.s002.pdf]

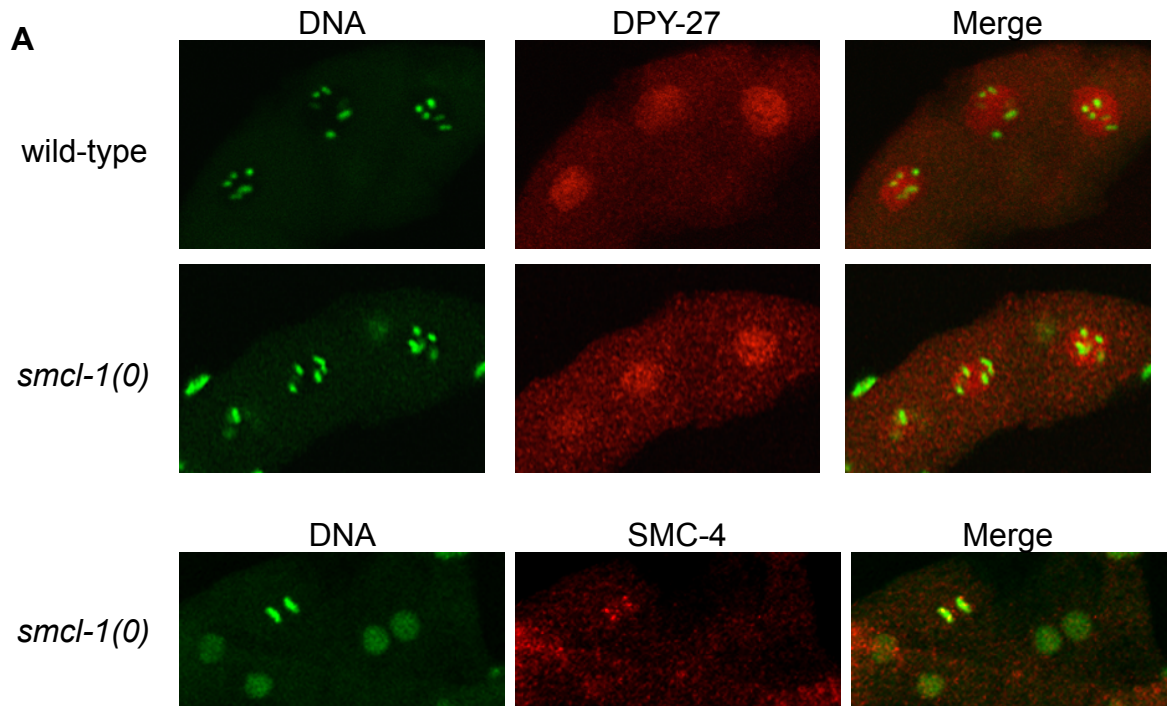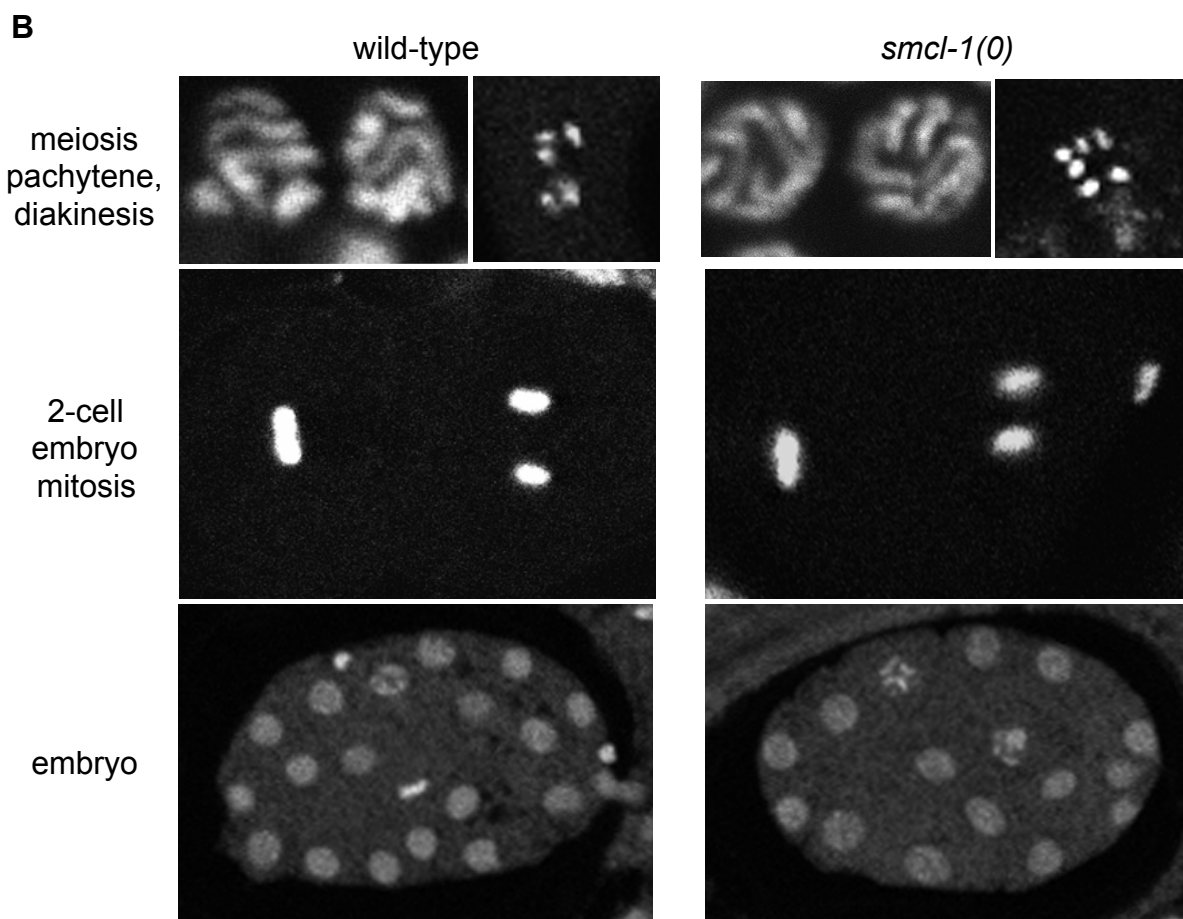

Supplement: S4 Fig — (A) DNA stain (green) and antibody against DPY-27 (red) in oocytes showing diffuse nuclear DPY-27 staining in both wild-type and smcl-1(0). Antibody against SMC-4 in smcl-1(0) embryo (bottom), showing typical localization to anaphase chromosomes [14]. (B) Comparison of chromosome morphology in wild-type and smcl-1(0) during meiotic pachytene and diakinesis (top), mitotic metaphase and anaphase in a 2-cell embryo (middle), and in early embryo interphase nuclei (bottom). (PDF) [file pgen.1006614.s004.pdf]

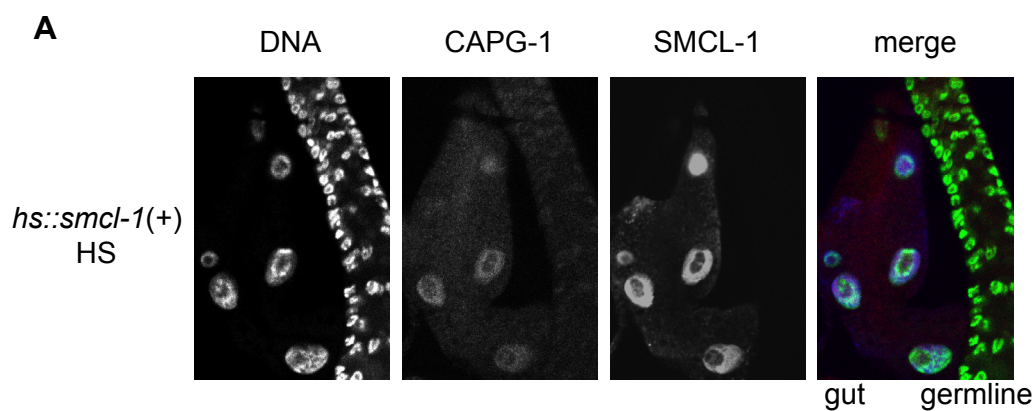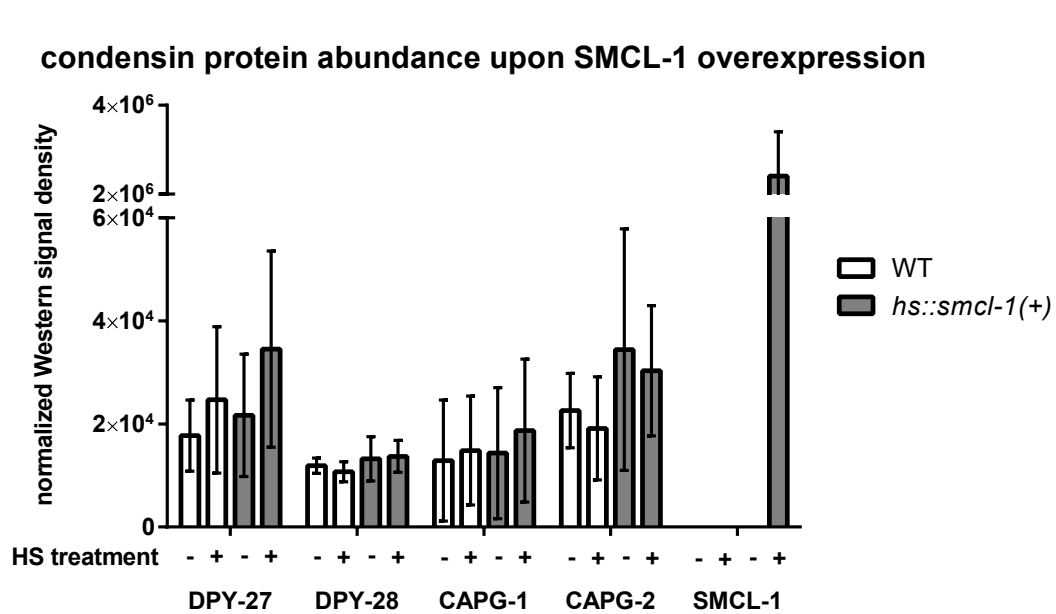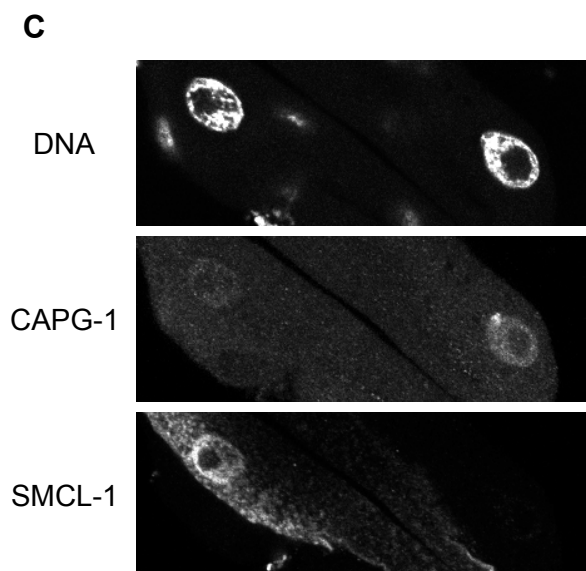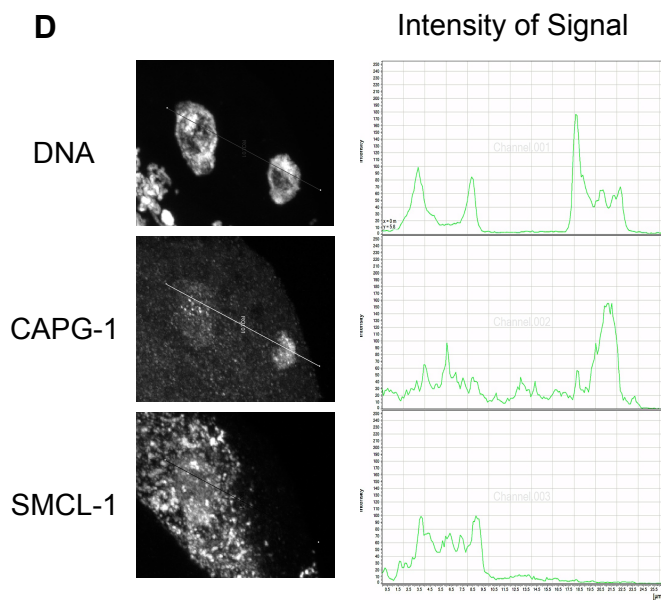

Supplement: S6 Fig — (A) Example of inducible hs:smcl-1(+) transgenic strain after heat shock, stained for DNA, CAPG-1, and SMCL-1, showing SMCL-1 overexpression is induced in the gut but not the germline. (B) Wild-type (white) or hs::smcl-1(+) (gray) young adults were subjected to 2 hours of heat shock (HS+, bottom) or no heat shock (HS-, bottom), recovered for 4 hours, then standard lysates prepared and analyzed on Western blots with antibodies against actin, the condensin subunits shown, and SMCL-1. Signals were quantified on an Odyssey CLx Imaging system and normalized using relative actin signal density. Mean values from 4 biological replicas are shown. No significant change in levels of each subunit was detected upon heat shock-induced SMCL-1 overexpression; p-values calculated by non-parametric Mann-Whitney test. Bars represent 95% confidence intervals. (C, D) Gut nuclei from the hs:smcl-1(+) mosaic strain after heat shock, stained for DNA (top), and immuno-stained for CAPG-1 (middle) and SMCL-1 (bottom). (C) Cell at right shows no SMCL-1 overexpression and CAPG-1 is localized in the nucleus with enrichment at a sub-nuclear focus (X chromosome). Cell at left shows SMCL-1 overexpression and lower levels of diffuse nuclear CAPG-1 staining and no X localization. Single confocal plane shown. (D) Stacked confocal images of another pair of nuclei (left) and quantification of pixel intensity (right) across a line (visible in CAPG-1 panel) drawn left to right, suggesting CAPG-1 is present but reduced in the SMCL-1 overexpressing nucleus. (PDF) [file pgen.1006614.s006.pdf]
