# Supplementary material for: An SMC-like protein binds and regulates Caenorhabditis elegans condensins
Source: PLoS Genet. 2017 Mar 16;13(3):e1006614. doi: 10.1371/journal.pgen.1006614 (PMC5373644; doi:10.1371/journal.pgen.1006614)
Supplement: S3 Fig — A set of species was examined for SMCL-1 like proteins based on SMC homology, intact Walker A motif, and non-conserved signature motif and Walker B motifs (see Methods, Results, and Fig 7). The left column shows proteins identified this way as well as a canonical SMC and SMCL-1 for reference; other columns indicate relevant protein features. Conserved motif sequences are shown below the motif labels in the first row. x represents any amino acid; h represents any hydrophobic amino acid; + represents amino acid in agreement with consensus. (PDF) [file pgen.1006614.s003.pdf]

**SMCL-1-like proteins**	**length**	**coiled coils**	**SMC hinge**	**Walker A GxxGxGK(S/T)**	**Signature motif LSGG(E/Q)(K/R)**	**Walker B hhhhDE**
**typical SMCs**	**1000-1500**	**yes**	**yes**	**++++++++**	**++++++**	**++++++**
**SMCL-1, C44C10.4**	**537**	**no**	**no**	**++++++++**	**FH++S+**	**+++++D**
CBG10266	639	no	no	+++A++++	F+++++	+++++N
CBG10265	305	no	no	+++S++++	F+++++	+++++N
CRE07623	334	no	no	++++++++	++++++	+++++N
CRE07629	347	no	no	++++++++	+++++S	+++++D
CBN26183	520	no	no	++++++++	MY+++Q	+++++D
CBN16092	529	yes	no	++++++++	++++++	+++++S
CBN08369	393	no	no	S++A++++	V+++++	+++++N
CBN06071	544	no	no	S+++++++	++++++	+++++N
Csp11_scaffold_629.g8681	525	yes	no	++++++++	++++++	+++++N
Csp5_scaffold_04032.g31882	330	no	no	++++++++		
Csp5_scaffold_04032.g31881	336				MG++++	+++++N
