# Supplementary material for: An SMC-like protein binds and regulates Caenorhabditis elegans condensins
Source: PLoS Genet. 2017 Mar 16;13(3):e1006614. doi: 10.1371/journal.pgen.1006614 (PMC5373644; doi:10.1371/journal.pgen.1006614)
Supplement: S5 Fig — (A) Brood size and viability phenotypes of hcp-6(mr17), a temperature-sensitive allele of a condensin II subunit, alone and in combination with the smcl-1 null allele. Top row shows results for shift from 15°C to 21.5°C at larval L1 stage, then brood counted from 3 hour egg lay of young adult. Middle shows results for shift from 15°C to 25°C at young adult stage, then total brood counted. Both are averages of two biological replicas, with >10 hermaphrodites of each genotype per replica. Bottom row shows results for shift from 15°C to 21.5°C at young embryo stage, then scored for percent of embryos laid that survive to hatch into L1 larvae, averaged from three replicas with >70 embryos per replica. (B) Average number of viable adult progeny per worm over its lifetime measured for the e428 allele of dpy-21, whose product is required for X dosage compensation but is not part of condensin IDC [67], alone or in combination with the smcl-1 null allele. (PDF) [file pgen.1006614.s005.pdf]

A

Phenotype	Genotype	
	hcp-6(mr17)	*hcp-6(mr17) ; smcl-1(0)*
Average brood size per worm (shift L1 to 21.5^{o}C)	17	20
Average brood size per worm (shift young adult to 25°C)	145	143
Embryo to L1 hatch rate (shift young embryos to 21.5^{o}C)	7%	7%

B

Phenotype	Genotype	
	dpy-21(e428)	*dpy-21(e428); smcl-1(0)*
Average viable adult progeny per worm	156	142
